# High-cell-density *Escherichia coli*–based nanobody production: benchmarking of subcellular targeting, strain background and process development strategies

**DOI:** 10.1186/s12934-026-03093-9

**Published:** 2026-08-29

**Authors:** Jan-Angelus Meyer, Marie Beneke, Sadija Altmüller, Alexander Reeb, Jannis Künzl, Nathalie Opilo, Theo Weise, Christos Gouloudis, Rebekka Biedendieck, Rainer Krull

**Affiliations:** 1 Institute of Biochemical Engineering, Technische Universität Braunschweig, Braunschweig, Germany; 2 Center of Pharmaceutical Engineering (PVZ), Technische Universität Braunschweig, Braunschweig, Germany; 3 Braunschweig Integrated Centre of Systems Biology (BRICS), Technische Universität Braunschweig, Braunschweig, Germany; 4 IBA Lifesciences GmbH, Göttingen, Germany; 5 Institute of Microbiology, Technische Universität Braunschweig, Braunschweig, Germany

**Keywords:** Nanobody production, VHH single-domain antibody, *Escherichia coli*, High-cell-density fed-batch cultivation, Recombinant protein production, Protein secretion, Bioprocess optimization, Design of experiments, Periplasmic expression, Cytoplasmic disulfide bond formation, Thermal protein stability

## Abstract

**Background:**

Nanobodies (NB) are compact single-domain antibody fragments with substantial diagnostic and therapeutic potential, but scalable microbial production under controlled bioreactor conditions remains insufficiently characterized. In this study a high-cell-density cultivation (HCDC) fed-batch process for an anti-human CD45 NB in *Escherichia coli* was established and evaluated how process parameters, host background, and subcellular targeting (cytoplasmic vs. periplasmic) affect yield and product quality including correct disulfide bond formation.

**Results:**

Using a two-stage design-of-experiments (DoE) strategy in 2 L stirred-tank bioreactors, OD_600nm_ at induction and post-induction temperature were identified as the main process variables controlling the post-purification nanobody concentration, whereas inducer concentration had no relevant effect within the tested range. Late induction at OD_600nm_ 125–150 combined with production temperatures around 21–22.5 °C defined a robust operating window and yielded more than 2.5 g L^−1^ purified nanobody after Strep-tag affinity chromatography. Benchmarking of five host strain–plasmid combinations under standardized HCDC conditions further increased NB yield indicating that production performance was directly governed by the combined host background and expression architecture. While *E. coli* NEBExpress® with periplasmic SP_PelB_ targeting reached around 2.4 g L^−1^ anti-hCD45 NB and the highest volumetric productivity (0.122 g L^−1^ h^−1^), *E. coli* SHuffle® T7 Express produced 2.2 g L^−1^ anti-hCD45 NB intracellularly. Nanobody-quality analysis revealed a pronounced difference between production routes. NB preparations obtained from periplasmic targeting constructs formed thermally uniform species with single nano differential scanning fluorimetry (DSF) unfolding transitions at 73.27–74.05 °C, whereas cytoplasmic derived preparations showed biphasic unfolding profiles with lower first transitions at 51.62–55.31 °C and second transitions at 68.30–71.53 °C indicating a higher thermal stability and also folding uniformity for secreted anti-hCD45 NB. Finally, flow cytometry confirmed functional binding of all tested anti-hCD45 NB to CD45-positive Jurkat cells.

**Conclusions:**

High-cell-density fed-batch cultivations of *E. coli* can reach > 2.5 g L^−1^ production of functional anti-CD45 nanobodies when induction timing, production temperature, host strain genetics, and subcellular location of the final product are aligned. The highest yield, most uniform, and thermally stable anti-hCD45 NB preparation was obtained with signal-peptide-mediated periplasmic targeting.

**Graphical Abstract:**

**Figure:**
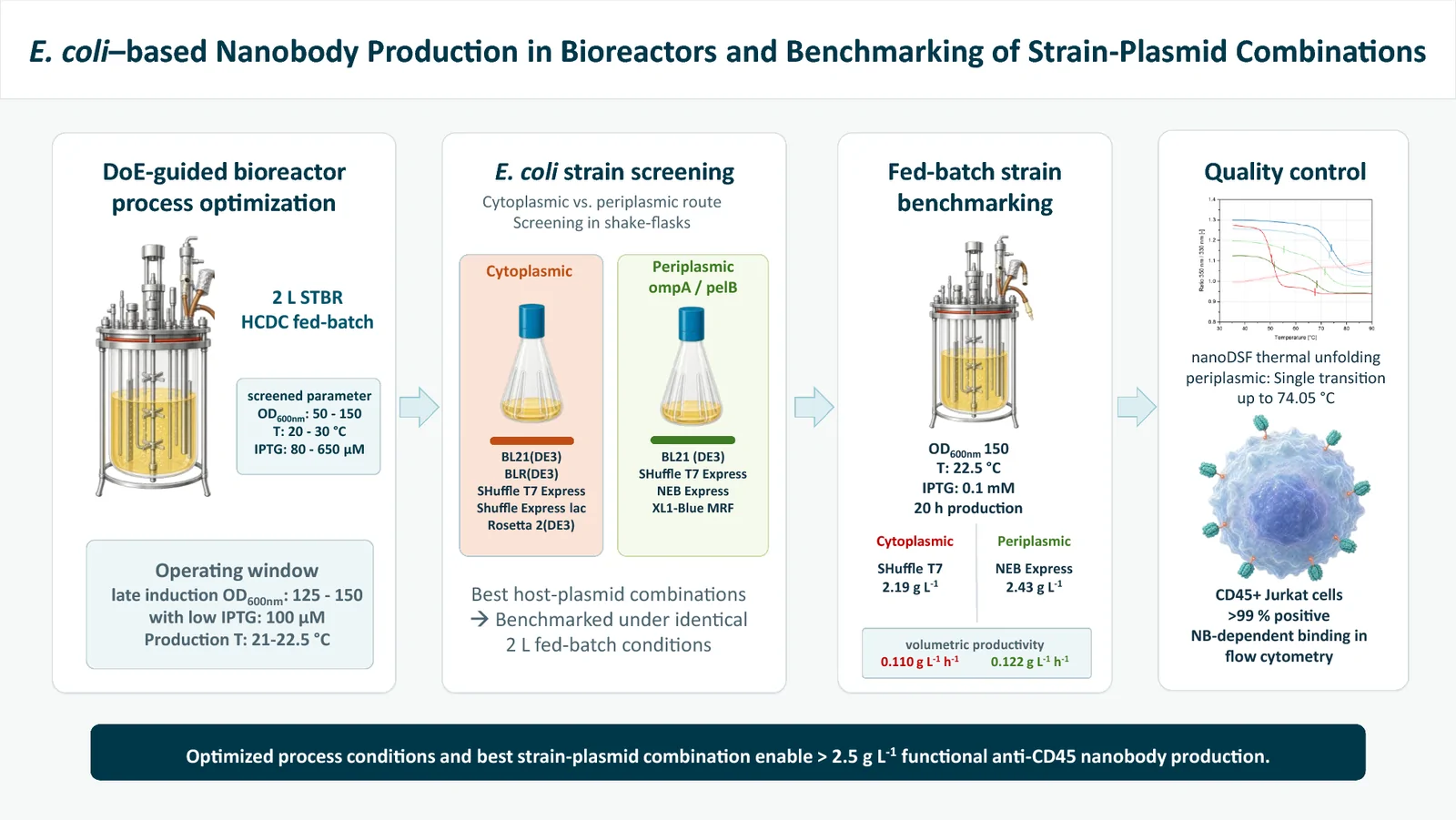

**Supplementary Information:**

The online version contains supplementary material available at https://doi.org/10.1186/s12934-026-03093-9.

## 1 Background

Nanobodies (NB), also referred to as VHH domains, originate from the antigen-binding variable domains of camelid heavy-chain antibodies, a naturally occurring antibody class that lacks light chains while retaining antigen-binding capacity [1]. Their single-domain architecture distinguishes them from conventional antibody formats and results in compact binding proteins of approximately 15 kDa that combine high specificity with favorable biochemical properties, including solubility, stability, modularity, and suitability for microbial production [2, 3]. In addition to these production-related advantages, the structural features of VHH domains, particularly their paratope geometry and often elongated complementarity-determining region, can enable recognition of epitopes that are less accessible to conventional antibodies [4, 5]. These properties have supported the increasing use of nanobodies as research reagents, diagnostic binders, and therapeutic molecules [6, 7].

The expanding application space of nanobodies creates a corresponding need for robust and scalable production platforms. Compared with full-length immunoglobulins, single-domain antibody fragments are structurally simpler and do not require assembly of separate heavy and light chains, making microbial production particularly attractive [3, 8, 9]. Among microbial hosts, *Escherichia coli* remains one of the most widely used expression systems because it grows rapidly, is genetically well characterized, offers extensive molecular toolkits, and can be operated at high cell densities in controlled cultivation processes [10–12]. However, high-level recombinant protein production in *E. coli* is not merely a question of transcriptional strength. Product yield and quality emerge from the interaction of expression rate, cellular resource allocation, folding capacity, redox environment, proteolysis, aggregation, and process conditions [10, 13, 14].

Correct folding is a central issue for proteins produced in *E. coli*, particularly when intramolecular disulfide bonds are required. For nanobodies and related antibody fragments, disulfide architecture can contribute to structural integrity, thermal stability, aggregation behavior, and antigen binding [15–18]. Because the reducing cytoplasm is generally unfavorable for spontaneous disulfide bond formation, recombinant production strategies commonly rely either on periplasmic export or on engineered cytoplasmic host backgrounds that support oxidative folding [15]. SHuffle^®^ strains (New England Biolabs, Ipswich, MA, USA) were developed to enable oxidative folding in the cytoplasm and support production of recombinant soluble proteins with correctly formed disulfide bonds [19]. Systematic host and folding-factor studies further show that disulfide-bonded antibody fragments can be produced in soluble form in the cytoplasm of engineered *E. coli*, however performance remains protein-, plasmid-, and host-dependent [20, 21].

Secretion to the periplasm offers a mechanistically distinct route. Proteins destined for the periplasm are commonly exported across the inner membrane by the general secretory (Sec-) pathway, where specific N-terminal signal peptides (SPs) mediate targeting and translocation in an unfolded, export competent state [22, 23]. This route is attractive for the recombinant production of antibody fragments because the periplasm naturally supports disulfide bond formation and can simplify recovery compared with intracellular location [8, 15]. However, Sec-dependent SPs are not universally transferable and efficient secretion depends on the SP itself, but also on translation initiation context, target-protein folding kinetics, expression level, and available translocation capacity [23–26]. For antibody fragments specifically, comparative studies of Sec- and signal recognition particle (SRP-) dependent SPs showed that choice of the SP can affect both display and soluble fragment production in *E. coli* [27]. Thus, cytoplasmic and periplasmic production must be evaluated as host-construct-process combinations rather than generic compartment choices.

Recombinant production also imposes a physiological burden on the host, arising from transcription, translation, folding, stress responses, impaired growth, or accumulation of the non-native proteins [13, 14]. In T7-RNA-polymerase (T7)-based *E. coli* systems, strong basal expression and transcriptional activity can become problematic for toxic, membrane-associated, aggregation-prone, or growth-burdening proteins [28]. Experimental dissection of recombinant protein burden has shown that transcription alone can measurably affect growth, while translation and downstream folding stress can further amplify this burden depending on the product [29]. Accordingly, high apparent expression does not necessarily translate into high amounts of soluble, correctly folded, functional nanobody; productive expression must match synthesis rate with folding and processing capacity [14, 24].

From a bioprocess perspective, cultivation scale and operating mode are equally important. Microtiter plates and shake flasks are valuable for early nanobody and antibody-fragment screening, but they only partially reflect controlled HCDC environments [30, 31]. Industrially relevant recombinant protein production in *E. coli* is commonly developed under fed-batch conditions, where substrate feeding, oxygen transfer, induction timing, temperature, and process duration shape biomass formation and product accumulation [12, 32, 33]. HCDC can improve volumetric productivity by increasing the amount of productive biomass, but it also intensifies demands on oxygen transfer, substrate control, heat removal, and cellular stress management [12, 32]. Consequently, parameters that appear secondary in small-scale screens can become decisive in bioreactors.

Temperature and induction strategy are particularly relevant. Lower production temperatures are frequently used to slow translation, reduce aggregation, and improve folding of recombinant proteins, whereas higher temperatures can favor rapid biomass formation but may increase folding stress or proteolytic loss [10, 13]. In fed-batch production of a recombinant VHH-based antivenom in *E. coli*, post-induction temperature was identified as a process parameter that affected product formation, supporting the relevance of temperature optimization for nanobody-related products at bioreactor scale [34]. Similarly, induction timing and inducer concentration influence how the production phase is balanced against biomass formation, metabolic state, and cellular capacity for product formation [12, 33].

Recent work illustrates the promise and the remaining knowledge gap in scalable nanobody production. Dynamic two-stage control strategies have enabled robust production and purification of nanobodies in *E. coli*, highlighting the value of coordinating growth and production phases rather than treating expression as a simple on/off event [35]. In parallel, fed-batch cultivation studies on disulfide-bonded proteins and antibody fragments show that host physiology and stress responses during production can shape process performance in ways that are not evident from endpoint production alone [21, 36]. Nevertheless, systematic datasets that connect HCDC process parameters, host-strain background, subcellular targeting route, purified nanobody yield, and product quality remain limited. This matters because nanobody production integrates recombinant expression strength, redox-dependent folding, signal-peptide-dependent export, metabolic burden, and process-controlled biomass formation.

Against this background, the present study addresses nanobody production in *E. coli* as an integrated microbial cell factory and bioprocess challenge. Rather than evaluating production only under screening-scale conditions, it investigates HCDC in stirred-tank bioreactors and links process optimization with host strain background and subcellular targeting architecture. As a model protein, an anti-human CD45 nanobody (IBA Lifesciences GmbH, Göttingen, Germany) was used, which contains an introduced disulfide bridge and therefore requires evaluation not only of production yield, but also of correct folding and retained target-binding function. The disulfide bond was introduced to enhance the conformational and thermal stability of the nanobody and to support the formation and maintenance of its correctly folded structure. By combining design-of-experiments (DoE)-based process parameter optimization with a comparative evaluation of cytoplasmic and periplasmic production strategies and subsequent product-quality assessment, this study aims to translate established molecular principles of recombinant production, oxidative folding, and protein export into a controlled bioreactor context relevant for subsequent scale-up.

## 2 Methods

### 2.1* E. coli* strains, expression vectors and genetic design

The *E. coli* strains used in this study are summarized in Table 1. All strains were selected to enable comparative evaluation of recombinant nanobody expression under different host backgrounds.

**Table 1:** *Escherichia coli* strains used in this study

Strain	Genotype	Relevant features	Source
*E. coli* BL21(DE3)	F^–^ *ompT gal dcm lon hsdS*_*B*_(r_B_^–^m_B_^–^) λ(DE3 [*lacI lacUV5*-*T7p07 ind1 sam7 nin5*])	T7 RNA polymerase under control of the *lac*UV5 promoter	Novagen (Merck KGaA, Darmstadt, Germany)
*E. coli* BLR(DE3)	F^–^ *ompT gal dcm lon hsdS*_*B*_(r_B_^–^m_B_^–^) λ(DE3 [*lacI lacUV5*-*T7p07 ind1 sam7 nin5*]) *(recA-srl)306::Tn10(Tet*^*R*^*)*	*recA*-deficient BL21(DE3) derivative	Novagen (Merck KGaA, Darmstadt, Germany)
*E. coli* XL1-Blue MRF′	*Δ(mcrA)*183 *Δ(mcrCB-hsdSMR-mrr)*173 *endA1 supE44 thi-1 recA1 gyrA96 relA1 lac [F’ proAB lacI*^*q*^*ZΔM15 Tn10 (Tet*^*R*^*)]*	Cloning host used for recombinant production of antibody-fragments before	Agilent Technologies (Santa Clara, CA, USA)
*E. coli* NEBExpress®	*huA2 [lon] ompT gal sulA11 R(mcr-73::miniTn10–*Tet^S^*)2 [dcm] R(zgb-210::Tn10–*Tet^S^*) endA1 Δ(mcrC-mrr)114::IS10*	Host optimized for high-level protein production	New England Biolabs (Ipswich, MA, USA)
*E. coli* SHuffle® Express	*fhuA2 [lon] ompT ahpC gal* λatt::pNEB3-r1-*cDsbC* (Spec^R^, *lacI*^*q*^*) ΔtrxB sulA11* R(*mcr-73::miniTn10*–Tet^S^)*2 [dcm] R(zgb-210::Tn10* –Tet^S^) *endA1 Δgor ∆(mcrC-mrr)114::IS10*	Allows cytoplasmic disulfide bond formation	New England Biolabs (Ipswich, MA, USA)
*E. coli* SHuffle® T7 Express	*fhuA2 lacZ::T7 gene1 [lon] ompT ahpC gal λatt::pNEB3-r1-cDsbC* (Spec^R^*, lacI*^*q*^) *ΔtrxB sulA11 R(mcr-73::miniTn10–*Tet^S^*)2 [dcm] R(zgb-210::Tn10 –*Tet^S^*) endA1 Δgor Δ(mcrC-mrr)114::IS10*	T7 RNA polymerase under control of the *lac*UV5 promoter, host allows cytoplasmic disulfide bond formation	New England Biolabs (Ipswich, MA, USA)
*E. coli* Rosetta™ 2 (DE3)	F^–^ *omp*T *gal dcm hsdS*_*B*_(r_B_^–^m_B_^–^) λ(DE3 [*lacI lacUV5*-*T7p07 ind1 sam7 nin5*]) pRARE (AUA, AGG, AGA, CUA, CCC, GGA, and CGG)	T7 RNA polymerase under control of the *lac*UV5 promoter, host supplemented with rare tRNAs	Novagen (Merck KGaA, Darmstadt, Germany)

All expression plasmids were based on a pET-28a backbone, which confers kanamycin resistance (KanR), contains the ColE1 origin of replication and encodes an anti-human CD45 NB fused to a C-terminal Twin-Strep-tag. In some plasmids the T7 promoter was exchanged against the tac promoter sequence based on the pMAL-vector system (New England Biolabs, Ipswich, MA, USA). For subcellular targeting, corresponding constructs were designed either for cytoplasmic production and for periplasmic export using N-terminal signal peptides of OmpA (SPOmpA) and PelB (SPPelB), respectively. All plasmids used in this study were provided by iba Lifesciences GmbH (Göttingen, Germany) and are summarized in Table 2.

**Table 2:** Expression plasmids used in this study

Plasmid ID	Promoter	Signal peptide
pT7-Cyto	T7	–
pT7-OmpA	T7	OmpA
pT7-PelB	T7	PelB
ptac-Cyto	tac	–
ptac-OmpA	tac	OmpA
ptac-PelB	tac	PelB

All plasmids encoded a C-terminally Twin-Strep-tagged anti-hCD45 nanobody and shared Kan^R^ and a ColE1 origin of replication; only variable plasmid features are shown

Plasmids were introduced into the respective *E. coli* strains by chemical transformation using CaCl₂-competent cells. Following incubation of plasmid DNA with competent cells on ice, a heat shock was applied at 42 °C. Transformed cells were subsequently regenerated in non-selective medium and plated on LB agar supplemented with kanamycin (50 mg L^−1^) for selection of transformants.

### 2.2 Shake-flask screening of new plasmid strains

An overnight preculture (12–14 h, 30 °C) of the corresponding strain in 50 mL Terrific Broth (TB-media, 12 g L^−1^ pancreatic digest of casein (peptone); 24 g L^−1^ yeast extract; 9.4 g L^−1^ dipotassium hydrogen phosphate; 2.2 g L^−1^ potassium dihydrogen phosphate; 4 g L^−1^ glycerol) supplemented with 50 mg L^−1^ kanamycin and 20 g L^−1^ glucose was directly inoculated from freshly transformed colonies on agar plates and grown aerobically at 30 °C. Precultures were used to inoculate 50 mL of the semi-defined medium in 500 mL baffled shake flasks to an initial OD₆₀₀ₙₘ of 0.1. The semi-defined medium consisted of defined mineral and buffer salts, vitamins, and trace elements, supplemented with yeast extract as the only complex medium component, 15 g L^−1^ glucose, and 50 mg L^−1^ kanamycin.

Cultivations were carried out at 30 °C and 150 min^−1^ (orbital shaker radius 5 cm). At an OD_600nm_ between 0.5 and 1.5, recombinant gene expression was induced by addition of 100 µM IPTG, followed by a temperature shift to 25 °C. The production phase was maintained for approximately 20 h. Cultures were subsequently harvested, final OD_600nm_ values were determined, and cells were collected by centrifugation. Cytoplasmic and periplasmic proteins were analyzed by qualitative sodium dodecyl sulfate (SDS) polyacrylamide gel electrophoresis (PAGE).

### 2.3 Cultivation of *Escherichia coli* in a 2 L stirred-tank bioreactor

2 L stirred-tank bioreactor (STBR) cultivations were conducted under controlled fed-batch conditions with constant feed rates of 20 mL h^−1^ to enable reproducible high-cell-density growth and defined induction of recombinant gene expression.

#### 2.3.1 Fed-batch strategy and cultivation phases

Precultures were grown overnight in 50 mL TB-medium with the addition of 20 g L^−1^ glucose and 50 mg L^−1^ kanamycin for a period of 8 to 10 h at a temperature of 30 °C and a shaking rate of 150 min^−1^ (rpm, orbital shaker radius 5 cm) in 50 mL shake flasks to an OD_600nm_ of approximately 10 to 15. The STBRs were inoculated with 8 to 12 mL of preculture achieving an initial OD_600nm_ of 0.1 in 1.2 L of semi-defined batch medium (defined mineral and buffer salts, vitamins, trace elements, supplemented with yeast extract) with the addition of 15 g L^−1^ glucose and 50 mg L^−1^ kanamycin. The cultivations were performed in parallelized 2 L STBRs (Getinge Livit Flex, Getinge AB, Gothenburg, Sweden). Following inoculation, cultivations were operated in batch mode at 30 °C and pH 6.8. The fed-batch phase was initiated upon depletion of glucose as indicated by a characteristic increase in pH or by glucose concentrations of less than 0.5 g L^−1^. The constant feeding process was initiated at a rate of 10–20 mL h^−1^. The feed comprised 800 mL of a solution containing 50% (w/v) glucose with 80 mM of MgSO₄ added as a supplement. At the target OD_600nm_ the recombinant gene expression was induced by the addition of IPTG at varying concentrations and the cultivation temperature was reduced according to the experimental design. The subsequent production phase was conducted as an induced fed-batch for precisely 20 h at a constant feed rate (20 mL h^−1^) and constant temperature. Table 3 shows all process parameters.

**Table 3:** Process parameters of cultivations in a 2 L stirred-tank bioreactor

Process parameter	Value
Temperature before induction	30 °C
Temperature after induction	20–30 °C
OD_600nm_ at induction	50–150
Minimum dissolved oxygen (DO) value	30%
pH	6.8
Stirring rate	500–1200 min^−1^
Air flow rate	1000–4000 mL min^−1^
O₂ flow rate	0–1000 mL min^−1^
Feed rate	20 mL h^−1^

The pH was maintained at 6.8 using a 25% (w/w) aqueous ammonia solution for pH control, without the addition of acid for negative regulation. The level of dissolved oxygen (DO) was maintained at 30% air saturation by employing a cascade control strategy that adjusted the stirring rate, the total gas flow rate, and the gas composition (compressed air and pure oxygen).

#### 2.3.2 Two-stage design of experiments and statistical analysis

A sequential two-stage DoE strategy was conducted using the established *E. coli* SHuffle^®^ T7 Express strain harboring pT7-Cyto. All DoE cultivations were performed in 2 L stirred-tank bioreactors as described in Sect. 2.3.1. The induced production phase was standardized to 20 h and operated at a constant feed rate of 20 mL h^−1^. The response variable was the concentration of affinity-purified NB, normalized to the original culture volume, 20 h after induction. Samples were taken 5, 10, and 20 h after induction to document the temporal progression of NB synthesis during the production phase.

To define appropriate factor ranges, data from the literature were consulted. For IPTG concentration, studies on recombinant protein production under high-cell-density conditions reported a relevant range of 80–650 µM [37, 38]. The selection of the temperature range was guided by literature indicating that reduced temperatures during the induction phase can enhance recombinant protein production, whereas higher temperatures primarily promote cell growth. Accordingly, a temperature range of 22.5–30 °C was investigated. As only limited literature data are available for the induction point in HCDC, OD_600nm_ values of 50, 100, and 150 were selected based on preliminary experiments to cover both early and late induction scenarios.

First, an eight-run fractional factorial screening design was generated in MODDE® (version 12.0.1; Sartorius Stedim Biotech, Göttingen, Germany) to evaluate the effects of IPTG concentration (80–650 µM), OD_600nm_ at induction (50–150), and post-induction temperature (22.5–30 °C) on NB concentration. Based on the screening outcome, OD_600nm_ at induction and post-induction temperature were selected for subsequent response-surface methodology (RSM), whereas IPTG concentration was fixed at 100 µM. Five additional experiments were generated using an onion D-optimal design, extending the investigated temperature range to 20 °C. The screening and RSM-extension datasets (Experiments I–XIII) were jointly evaluated using a quadratic response-surface model.

Model performance was assessed using R2, Q2, residual standard deviation, model validity, reproducibility, and residual diagnostic plots. Regression coefficients were interpreted using 90% confidence intervals at an exploratory significance level of α = 0.1.

#### 2.3.3 Process monitoring and sampling during bioreactor cultivation

During STBR cultivation, process parameters were continuously monitored using online sensors. The pH was measured using a pH probe (Hamilton Company, Bonaduz, Switzerland). Dissolved oxygen concentration (DO) was recorded using a DO probe (Hamilton Company, Bonaduz, Switzerland). Optical density was monitored online using a combined transmission and reflection probe (Hamilton Dencytee, Hamilton Company, Bonaduz, Switzerland), enabling non-invasive, real-time biomass trend monitoring directly in the STBR. Online OD signals were correlated to offline OD_600nm_ measurements obtained by photometric analysis.

Offline analyses were performed using discrete samples and served as reference measurements for online monitoring. OD_600nm_ was determined photometrically using a Libra S11 instrument (Biochrom GmbH, Schaffhausen, Switzerland). Biomass concentration was quantified as cell dry weight (CDW) using a halogen moisture analyzer (MBG series, VWR International GmbH, Darmstadt, Germany), and cell wet weight (CWW) was determined gravimetrically. Glucose concentrations in culture samples were measured using a glucose analyzer (YSI 2500, Kreienbaum NEO GmbH, Langenfeld, Germany).

During the production phase, 50 mL samples were withdrawn at defined time points (0, 5, 10, and 20 h after induction) for determination of NB concentration; subsequent sample handling and cell disruption are described in Sect. 2.3.4.

#### 2.3.4 Sample handling and preparation for downstream analysis

In addition to samples for determination of OD, CWW and CDW, a sample of 50 mL was collected from each culture starting after induction. Cells were harvested by centrifugation for 15 min at 10,000×*g* at 20 °C. The supernatant was used for determination of glucose concentration and for SDS–PAGE analysis to assess secreted proteins. The sedimented cells were weighed for determination of CWW and subsequently resuspended in Tris–HCl buffer (100 mM Tris-HCl, 150 mM NaCl, 1 mM EDTA, pH 8). To enable direct comparison across all production routes, harvested cell pellets were subjected to whole-cell disruption with a CWW concentration of approximately 250 g L^−1^ using an ultrasonic homogenizer (Digital Sonifier W-250 D, Branson, Missouri, USA) operated at 30% amplitude and a frequency of 20 kHz for a total sonication time of 18 min. Sonication was performed in a beaker placed in an ice bath to limit sample heating. Following cell disruption, cell debris was removed by centrifugation at 25,000×*g* for 20 min at 15 °C. The resulting supernatant was sterile-filtered and kept at 4 °C until subsequent Strep-tag affinity chromatography. No selective periplasmic fractionation was performed.

### 2.4 Nanobody purification by Strep-tag affinity chromatography and quantification

NB were purified from sterile-filtered cell lysates by Strep-tag-based affinity chromatography. Chromatographic separations were performed on an ÄKTA pure™ chromatography system (Cytiva, Marlborough, MA, USA) equipped with a Strep-Tactin^®^XT 4Flow^®^ high-capacity column (IBA Lifesciences GmbH, Göttingen, Germany). Prior to sample loading, the system and column were equilibrated with Tris–HCl buffer (100 mM Tris-HCl, 150 mM NaCl, 1 mM EDTA, pH 8) according to the manufacturer’s recommendations. Sterile-filtered cell lysates were injected via a Cytiva Superloop (10 mL). No analytical determination of total protein or NB concentration was performed before column loading. Instead, preliminary affinity-chromatography runs were initiated with 5 mL of sterile-filtered lysate. The loading volume was subsequently adjusted iteratively to avoid substantial under- or overloading and to approximate 80–90% of the estimated column binding capacity. The empirically established loading volume was then applied for the triplicate purifications. After sample application, unbound proteins were removed by washing with Tris–HCl buffer. Strep-tagged NB were subsequently eluted using elution buffer (100 mM Tris-HCl, 150 mM NaCl, 1 mM EDTA, 50 mM biotin, pH 8). System operation, method control, and data acquisition were carried out using UNICORN™ software. All NB samples were purified according to this protocol and plotted in the respective diagrams as NB concentrations (in g L^−1^).

All fractions were analyzed by SDS–PAGE (13.5% acrylamide). Fractions containing nanobody as identified by online UV absorbance at 280 nm (extinction coefficient of 0.497 mL mg^−1^ cm^−1^) recorded on the ÄKTA system and SDS–PAGE analysis were pooled. Protein concentration of the pooled NB fraction was determined spectrophotometrically using a NanoDrop™ instrument (Thermo Fisher Scientific, Waltham, MA, USA).

### 2.5 Protein analytics and functional characterization

Purified nanobodies were analyzed to confirm protein identity and to assess structural integrity and functionality. Initial analytical characterization was performed by SDS–PAGE followed by Western blotting. Analytical methods included SDS–PAGE, Western blotting, nano differential scanning fluorimetry (nanoDSF), and immunofluorescence flow cytometry for binding activity assessment.

#### 2.5.1 Western blot analysis

Western blot analysis was performed for detection of Strep-tagged nanobody variants after purification. Equal amounts of Strep-tagged affinity-purified anti-hCD45 NB treated with SDS for denaturing and 2-mercaptoethanol for reducing the disulfide bonds were separated by SDS–PAGE (13.5% acrylamide), transferred onto a polyvinylidene fluoride (PVDF) membrane, and immunodetected using Strep-Tactin^®^ AP against the Strep-tag (IBA Lifesciences GmbH, Göttingen, Germany). For getting a sufficient concentration of purified anti-hCD45 NB recombinantly produced with BL21(DE3) pT7-Cyto a concentration step using Amicon^®^ Ultra Centrifugal Filter, 10 kDa MWCO (Merck, Millipore, Darmstadt, Germany) was necessary before loading. The apparent molecular weight of the detected bands was used to assess correct processing of the NB.

#### 2.5.2 Thermal stability analysis by nano differential scanning fluorimetry (nanoDSF)

Thermal stability of purified NB was assessed by nano differential scanning fluorimetry (nanoDSF) using a Prometheus instrument (NanoTemper Technologies GmbH, Munich, Germany). Samples were prepared in phosphate-buffered saline (PBS, pH 7.4; 8.0 g L^−1^ sodium chloride; 0.2 g L^−1^ potassium chloride; 1.44 g L^−1^ disodium hydrogen phosphate; 0.245 g L^−1^ potassium dihydrogen phosphate) and loaded into standard nanoDSF capillaries. Intrinsic fluorescence emission at 330 and 350 nm was recorded during a controlled heating ramp and a subsequent cooling phase. The 350/330 nm fluorescence ratio was used to monitor thermally induced unfolding and potential refolding.

#### 2.5.3 Immunofluorescence flow cytometry for binding activity assessment

Binding activity of the anti-human CD45 NB was assessed by immunofluorescence flow cytometry using Jurkat ACC-282 cells (Leibniz Institute DSMZ-German Collection of Microorganisms and Cell Cultures GmbH, Braunschweig, Germany), a human leukocyte cell line expressing CD45 on the cell surface. Cells were transferred into PBS (pH 7.4; 8 g L^−1^ sodium chloride; 200 mg L^−1^ potassium chloride; 1.44 g L^−1^ disodium hydrogen phosphate; 245 mg L^−1^ potassium dihydrogen phosphate) supplemented with 10 mM EDTA and incubated with the Strep-tagged anti-hCD45 NB. NB binding was detected via fluorescent labeling using streptavidin conjugated to R-phycoerythrin (PE) (Strep-Tactin^®^XT PE; IBA Lifesciences GmbH, Göttingen, Germany), which binds specifically to the Strep-tag without interfering with antigen recognition. As a positive control for CD45 binding, a PE-labeled monoclonal mouse anti-hCD45 mAb was used. Fluorescent staining was performed according to the manufacturer’s protocol for fluorescent labeling with Strep-Tactin^®^XT PE. Labeled cells were analyzed under standard operating conditions with a CytoFLEX flow cytometer (Beckman Coulter, Brea, CA, USA). Flow-cytometric data were evaluated by sequential gating, in which debris and cell aggregates were excluded based on forward- and side-scatter properties before retaining morphologically intact single-cell Jurkat events; no additional viability dye or live/dead stain was used. NB-dependent binding was then assessed within this gated single-cell population by PE fluorescence, using Q1-UR as the upper-right fluorescence-positive quadrant and median PE-A as the fluorescence intensity readout.

## 3 Results and discussion

The results and discussions are presented in the order of process development (Sect. 3.1), comparison of production systems (Sect. 3.2), and product characterization (Sect. 3.3). First, HCDC process was optimized using a DoE approach. Subsequently, selected cytoplasmic and periplasmic *E. coli* expression systems were compared under standardized bioreactor conditions. Finally, the purified NB were characterized with respect to their identity, thermal behavior, and CD45-binding functionality.

### 3.1 Process optimization for high-cell-density nanobody production using a Design of Experiments (DoE) approach

Design of experiments (DoE) enables the simultaneous evaluation of multiple process factors and their interactions with a limited number of experiments [39]. The two-stage DoE described in Sect 2.3.2 was used to identify a robust operating window for high-cell-density NB production.

**Fig. 1:**
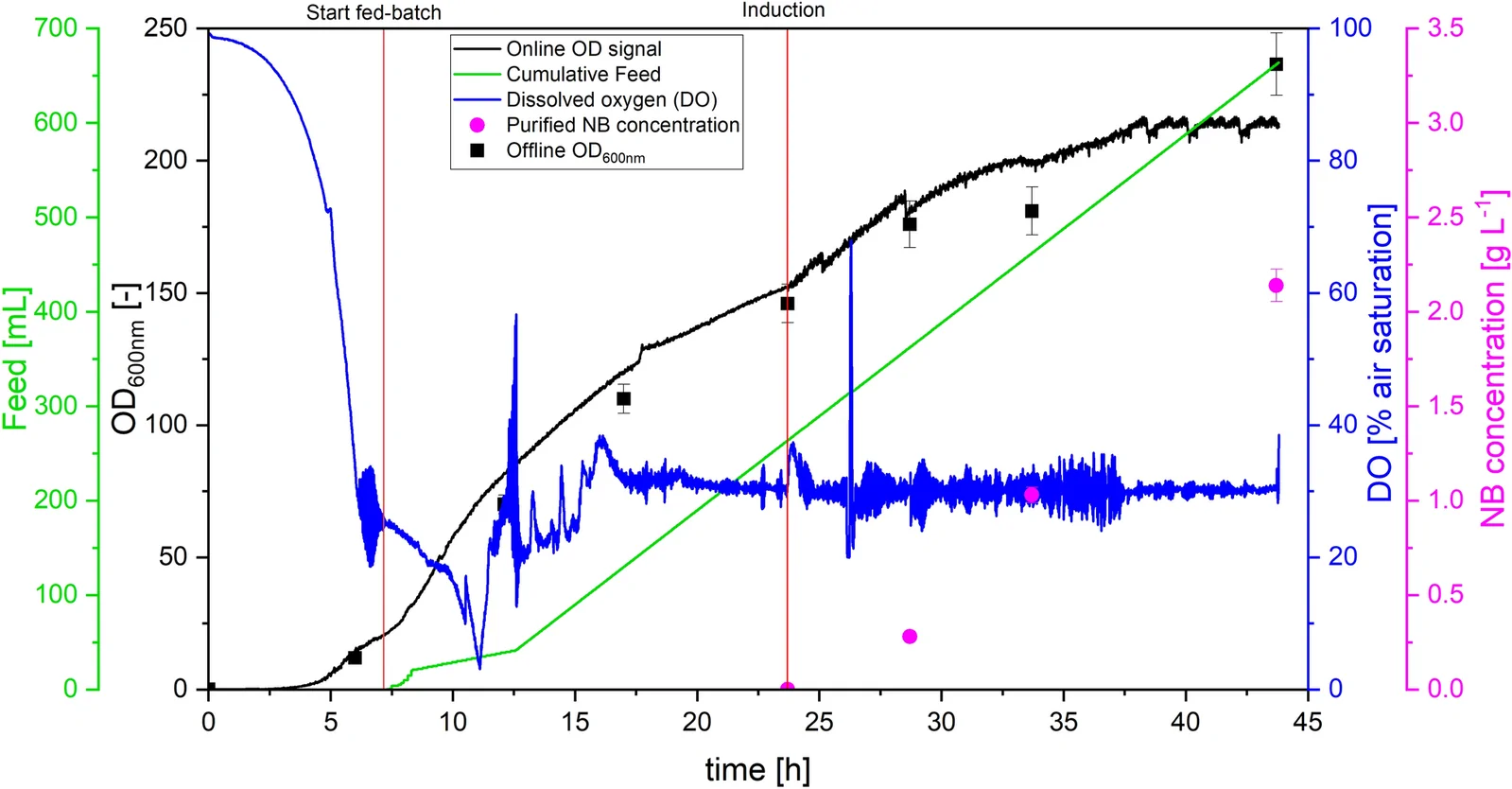
Representative 2 L fed-batch cultivation of *E. coli* SHuffle® T7 Express harboring the pT7-Cyto plasmid (conditions see Sect. 2.3). Induction was performed with 100 µM IPTG at OD₆₀₀ₙₘ = 150, followed by a temperature shift from 30 to 22.5 °C. pH setpoint was 6.8. A constant feed rate of 20 mL h^−1^ was applied from induction onwards. Shown are the online turbidity signal (Hamilton Dencytee in situ probe), offline OD₆₀₀ₙₘ measurements, dissolved oxygen (DO), cumulative feed addition and total NB concentration after whole-cell disruption and purification at the sampling time points 0, 5, 10 and 20 h after induction. This cultivation was subsequently used as a validation of the robust operating point derived from the DoE described in Sect. 3.1.2. The validated operating conditions were subsequently applied to the comparative cultivations described in Sect. 3.3

To illustrate the standardized 2 L fed-batch workflow at the selected operating point, a representative confirmatory cultivation profile is shown (Fig. 1), including online turbidity (Hamilton Dencytee *in situ* probe), offline OD_600nm_, cumulative feed addition, dissolved oxygen (DO), and post-purification NB concentration at the sampling time points.

The representative fed-batch cultivation showed stable dissolved-oxygen control throughout the growth and production phases. Feeding was initiated after approximately 7.5 h at 5–10 mL h^−1^ and increased to a constant feed rate of 20 mL h^−1^ from 12 h onward. From approximately 12 h until induction, oxygen demand was covered by cascade-controlled supplementation with pure oxygen (50–800 mL min^−1^). Production of recombinant anti-hCD45 NB was induced after approximately 24 h with 100 µM IPTG, concomitant with a temperature shift from 30 to 22.5 °C. Biomass accumulation slowed after approximately 28 h, whereas purified nanobody concentration continued to increase, reaching 2.14 g L^−1^ at the end of cultivation.

#### 3.1.1 Screening of key process parameters by fractional factorial DoE

At the beginning of the DoE, a factor screening was conducted to characterize the parameters and identify the key variables that influence the selected response. Results from the eight-run screening design are summarized in Table 4 (Experiments I-VIII).

**Table 4:** DoE experimental matrix and response for 2 L fed-batch bioreactor cultivations

	Experiment	OD₆₀₀ₙₘ at induction [–]	Post-induction temperature [°C]	IPTG concentration [µM]	NB concentration [g L^−1^]
**Screening phase**	I	150	30.0	650	0.80 ± 0.04
	II	125	27.5	250	0.64 ± 0.02
	III	100	25.0	100	0.99 ± 0.03
	IV	100	26.3	370	0.70 ± 0.03
	V	50	22.5	650	1.00 ± 0.06
	VI	50	30.0	80	0.47 ± 0.02
	VII	100	26.3	370	0.98 ± 0.03
	VIII	150	22.5	80	1.59 ± 0.02
**Addition to the RSM phase**	IX	125	24.0	100	2.85 ± 0.06
	X	150	22.5	100	2.84 ± 0.05
	XI	100	20.0	100	1.80 ± 0.02
	XII	80	27.5	100	2.51 ± 0.06
	XIII	150	20.0	100	2.41 ± 0.07
**Confirmatory run (Fig. **1**)**	-	150	22.5	100	2.14 ± 0.04

All *E. coli* cultivations were performed according to Sect. 2.3. Experiments I–VIII correspond to the fractional factorial screening DoE (Sect. 3.1.1). Experiments IX–XIII extended this initial design for subsequent response surface methodology (RSM) using an onion D-optimal design (Sect. 3.1.2). The response was the concentration of affinity-based purified NB (see Sect. 2.4) 20 h after induction. OD₆₀₀ₙₘ at induction is reported with an uncertainty of ± 5; temperature and IPTG concentration were applied without relevant experimental uncertainty. The confirmatory run represents the cultivation shown in Fig. 1, performed independently at the DoE-derived operating point to assess the suitability of the selected conditions for subsequent process development; it was not included in model fitting.

The factor screening revealed a pronounced dependence of the final product titer on the investigated process parameters. The lowest titer (0.47 g L^−1^) was obtained in Experiment VI, in which induction was carried out with 80 µM IPTG at an OD_600nm_ of 50 followed by cultivation at 30 °C. In contrast, the highest final titer of 1.59 g L^−1^ was achieved in Experiment VIII, which also employed 80 µM IPTG, but at a substantially higher OD_600nm_ of 150 at induction and a reduced cultivation temperature of 22.5 °C.

**Fig. 2:**
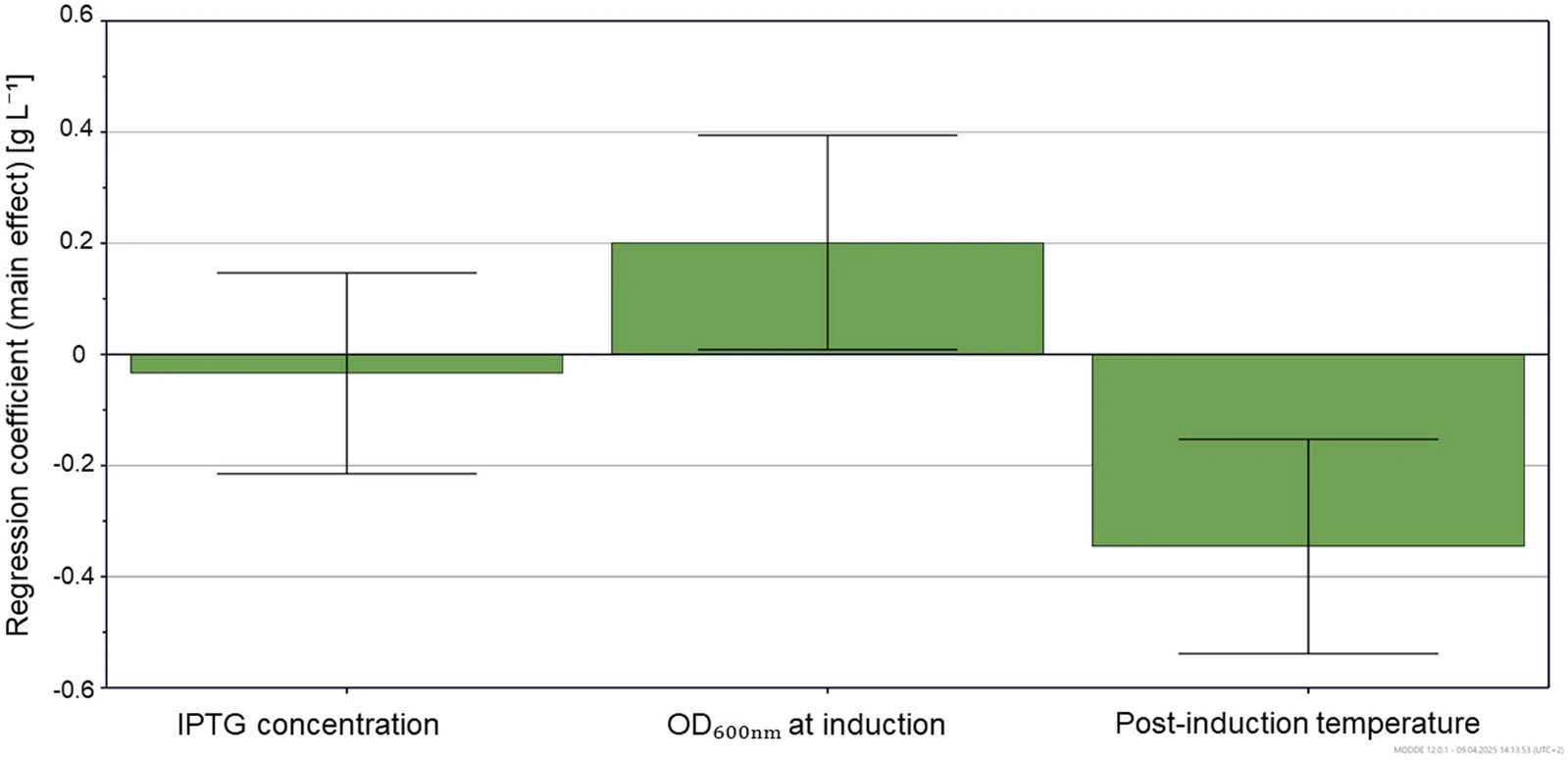
Coefficient plot (fractional factorial screening DoE, MODDE®). Estimated main effects of IPTG concentration, OD₆₀₀ₙₘ at induction, and post-induction temperature on NB concentration (g L^−1^) determined 20 h after induction. Error bars indicate 90% confidence intervals (R2 = 0.828; Q^2^ = 0.460; RSD = 0.189 g L^−1^; model validity = 0.890; reproducibility = 0.658)

The statistical analysis of the experimental data was performed with MODDE® using a linear main-effects screening model, which is typically applied in fractional factorial designs involving a limited number of experiments for parameter characterization. This model enables the assessment of the linear effects of the investigated factors, while potential interaction effects cannot be resolved at the screening level. The resulting coefficient plot (Fig. 2) illustrates the linear effects of the individual factors. Positive or negative coefficients indicate the extent to which a parameter increases or decreases the NB concentration. To assess statistical significance, the corresponding confidence intervals were evaluated, effects were deemed significant if their confidence intervals did not intersect the zero line.

With a coefficient of determination of R2 = 0.828 and Q^2^ = 0.460, the model indicated that OD_600nm_ at the time of induction exerted a significant positive effect on the product titer. This observation is consistent with literature reports demonstrating that higher optical densities at induction can positively influence both product titer and space–time yield, as a larger fraction of productive biomass is available [40].

A reduction in cultivation temperature during the production phase also showed a significant positive effect on nanobody titer. This finding corroborates previous studies reporting enhanced yields of recombinant proteins and nanobodies at lower induction temperatures, whereas increasing temperatures were associated with reduced product titers [34]. In contrast, IPTG concentration did not exhibit a significant effect on product titer within the investigated parameter range. As the inducer represents one of the most cost-intensive process components, this finding indicates a relevant potential for reducing overall process costs.

In summary, the DoE screening phase primarily served to identify and narrow down relevant process parameters rather than to determine a global optimum. The results demonstrated that high OD_600nm_ values at induction and lower cultivation temperatures during the production phase have a significant positive impact on nanobody titer. Moreover, no significant interaction effects between the examined parameters were identified at the screening level. Based on these findings, the significant parameters were subsequently subjected to targeted optimization using a response-surface model to determine their optimal combination for maximizing product concentration in the subsequent optimization step.

#### 3.1.2 Response surface modeling (Onion D-optimal design)

Following the screening phase, a response surface methodology (RSM) optimization phase was conducted to further evaluate the previously identified key factors [41]. From the screening (Sect. 3.1.1), OD_600nm_ at induction and the production-phase temperature were identified as the key factors. As the inducer concentration showed no effect on purified product concentration within the investigated range, all experiments were induced with a constant IPTG concentration of 100 µM. The post-induction temperature range was extended towards values down to 20 °C, to examine whether a further temperature reduction could additionally increase product concentration [42]. The additional experiments performed in the optimization phase (Experiments IX–XIII) and the resulting post-purification NB concentrations are summarized in Table 4. As these experiments expanded the design space defined during screening, datasets from both phases were jointly evaluated for subsequent modeling.

Experiments IX and X yielded the highest concentrations of purified NB of the entire DoE, reaching 2.85 and 2.84 g L^−1^, respectively. In Experiment IX, induction was performed at OD_600nm_ = 125 followed by a temperature shift to 24 °C, while in Experiment X, induction was performed at OD_600nm_ = 150 followed by a shift to 22.5 °C. These results corroborated the screening-derived trend (Sect. 3.1.1) that a high OD_600nm_ at induction in combination with a low production-phase temperature favor a high concentration of purified NB. At the same time, the data indicated that temperature cannot be reduced indefinitely: in the direct comparison of Experiments X and XIII, which differed only in the production-phase temperature, a further reduction to 20 °C resulted in a lower concentration of purified NB (Table 4).

**Fig. 3:**
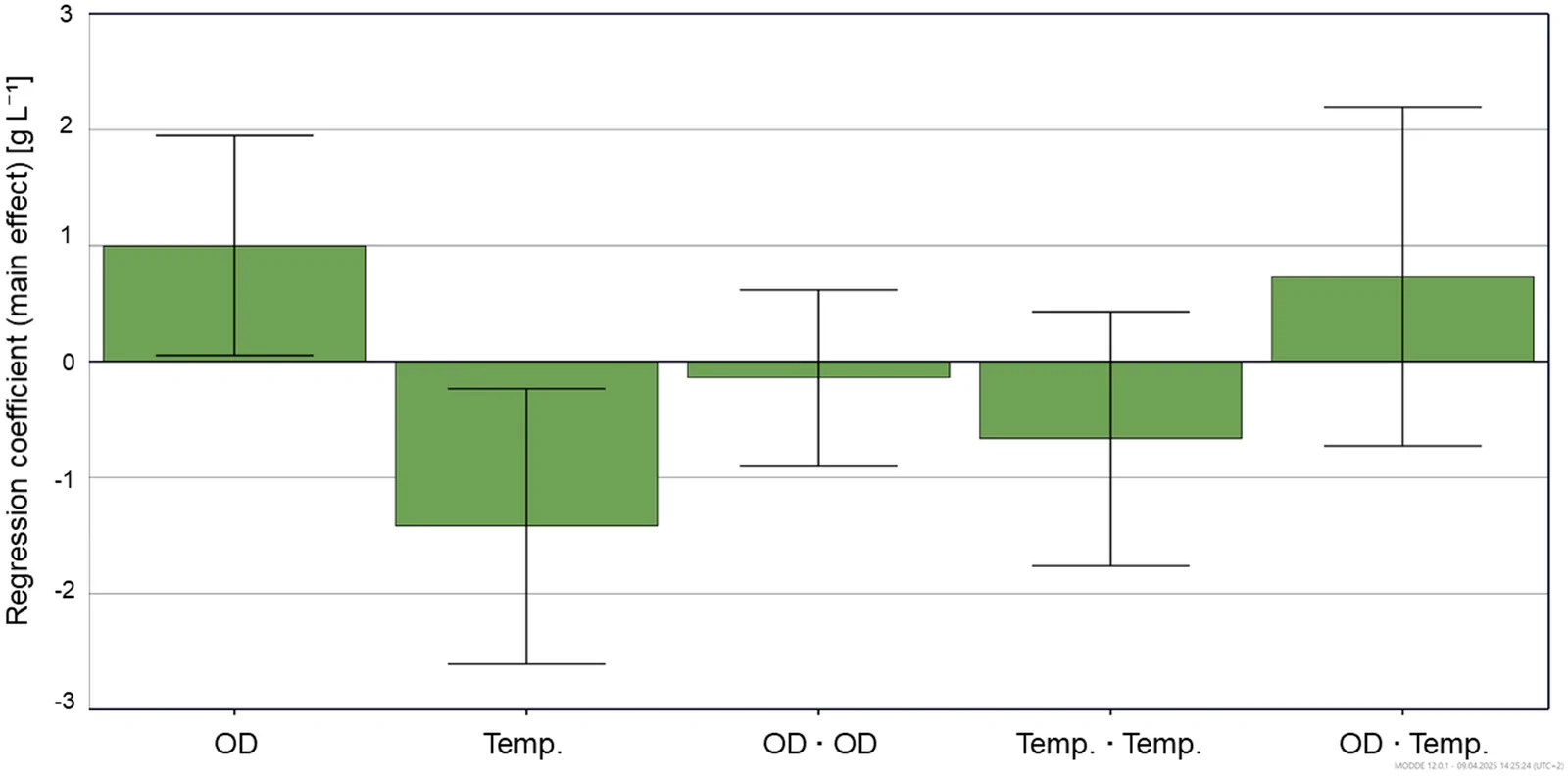
Coefficient plot (MODDE®) showing the estimated linear, quadratic, and interaction effects of OD₆₀₀ₙₘ at induction and post-induction temperature on NB concentration (g L^−1^) determined 20 h after induction (combined screening + optimization dataset; n = 13). Bars represent regression coefficients; whiskers indicate 90% confidence intervals. The model showed an adequate fit to the experimental data (R2 = 0.784; Q^2^ = 0.510; RSD = 0.204 g L^−1^; model validity = 0.813; reproducibility = 0.606)

Statistical analysis of the combined datasets (screening + optimization; n = 13) in MODDE® confirmed the observed trends and indicated an adequate fit to the experimental data with R2 = 0.784 and Q^2^ = 0.510 (Fig. 3). Model performance was assessed using MODDE® diagnostics: no significant lack-of-fit was observed (p = 0.518), and residual plots showed no major deviations from model assumptions (details including cross-validation are provided in the Supplementary Information). As shown in Fig. 3 (coefficient plot), the coefficients suggested a positive effect of higher OD_600nm_ values at induction and a negative effect of higher temperatures during the production phase (within the exploratory significance level α = 0.1). The quadratic terms of the onion D-optimal design did not indicate curvature for OD_600nm_, suggesting a predominantly linear relationship between OD_600nm_ and purified product concentration within the investigated range; a comparable linear relationship was also reported by Wechselberger et al. [40]. For temperature, the quadratic term suggested a slight tendency towards nonlinearity, indicating a potential temperature optimum during the production phase. This was supported by the comparison of Experiments X and XIII: decreasing the temperature by 2.5–20 °C resulted in a 15% lower purified nanobody concentration. Mechanistically, elevated temperatures can promote cellular stress, protein aggregation, and proteolytic degradation [10, 34]. Conversely, lower temperatures reduce cellular growth and translation rates; at the lower end of the investigated temperature range, the temperature downshift may additionally induce cold-shock responses that alter ribosome function and translational activity [10, 43].

**Fig. 4:**
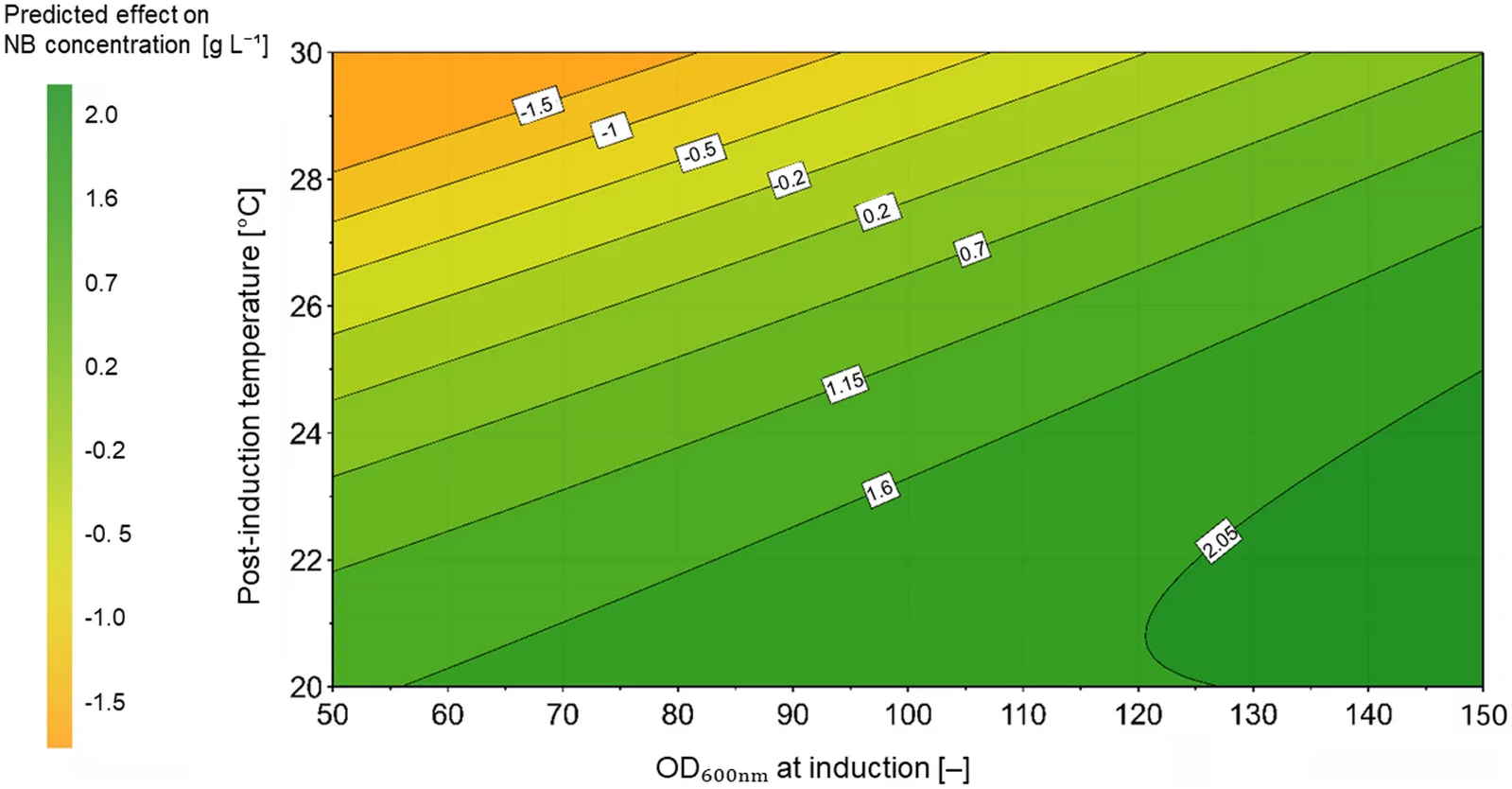
Response surface plot (MODDE®) from the RSM (Onion D-optimal design) showing the model-predicted NB concentration (g L^−1^) as a function of OD600nm at induction and post-induction temperature (IPTG fixed at 100 µM). The model predicts increasing NB concentration with higher OD_600nm_ and lower temperatures, with diminishing gains below approximately 21–22.5 °C, supporting selection of a robust operating window at OD_600nm_ between 125 and 150, and 21–22.5 °C, respectively

The interaction term between OD_600nm_ and temperature after induction was not significant (Fig. 4). However, the data indicated a combined effect of the parameter combination: While Experiment V (OD_600nm_ at induction = 50) at 22.5 °C achieved a NB concentration of 1.0 g L^−1^ (Table 4), Experiment X at the same temperature with OD_600nm_ at induction of 150 achieved a NB concentration that was nearly threefold higher (2.84 g L^−1^) (Table 4). Accordingly, the RSM plot (Fig. 4) shows increasing NB concentration with increasing OD_600nm_ and decreasing temperature, although the increases diminish at temperatures below approximately 21–22.5 °C.

In summary, the optimization phase confirmed the screening results and literature-derived expectations that high OD_600nm_ values at induction and low production-phase temperatures increase purified NB concentration. The model analysis suggested a predominantly linear relationship between OD_600nm_ and purified NB concentration and indicated a temperature optimum in the range of approximately 21–22.5 °C. The comparison of Experiments X and XIII showed that a further decrease to 20 °C reduced purified NB concentration. Based on the combined datasets (screening + optimization), a working operating point for subsequent process steps was defined as late induction at OD_600nm_ = 125 – 150, a temperature shift in the production phase to 21–22.5 °C, and 100 µM IPTG. An independent confirmatory run performed under these conditions yielded a purified NB concentration of 2.14 g L^−1^ after 20 h, as reported in Table 4, and its representative process profile is shown in Fig. 1. The suitability of this operating point for further process development is additionally supported by the subsequent cultivations performed under these conditions (Sect. 3.3).

### 3.2 Shake-flask screening of different combinations of *E. coli* hosts and expression plasmids

To identify the most suitable production hosts prior to conducting detailed bioreactor studies, a preliminary qualitative screening was first performed in shake flasks. A selection of seven *E. coli* strains was combined with six expression plasmids, encompassing cytoplasmic and periplasmic expression strategies (see Table 5 for details).

**Table 5:** Shake-flask screening of E. coli host–plasmid combinations

	BL21(DE3)	BLR(DE3)	XL1-Blue MRF′	NEB Express®	SHuffle® T7 Express	SHuffle® Express	Rosetta™ 2(DE3)
pT7-Cyto	cytoplasmic OD 23.90 ± 0.90	cytoplasmic OD 13.55 ± 1.25	–	–	**cytoplasmic OD 24.95 ± 0.65**	–	cytoplasmic OD 19.15 ± 0.65
ptac-Cyto	–	–	–	–	–	**cytoplasmic OD 25.15 ± 0.55**	–
pT7-OmpA	periplasmic OD 20.55 ± 0.95	–	–	–	periplasmic OD 22.45 ± 1.15	–	–
ptac-OmpA	–	–	periplasmic OD 8.70 ± 1.69	**periplasmic OD 28.45 ± 0.25**	–	–	–
pT7-PelB	periplasmic OD 21.50 ± 1.00	–	–	–	periplasmic OD 23.95 ± 0.65	–	–
ptac-PelB	–	–	periplasmic OD 6.45 ± 0.35	**periplasmic OD 28.90 ± 0.20**	–	–	–

Entries indicate the intended NB localization and OD_600nm_ at harvest (mean ± SD, n = 2). Bold-highlighted combinations showed robust growth and a distinct NB-associated band at approximately 17 kDa relative to the plasmid-free host control. A dash indicates a combination not tested because of promoter–host incompatibility. NB production was assessed qualitatively by SDS-PAGE of total cell-associated protein after whole-cell disruption; isolated periplasmic fractions and culture-broth NB concentrations were not determined.

The objective of the screening was to rapidly assess detectable nanobody expression by SDS–PAGE and basic growth robustness under standard shake-flask conditions.

In the case of *E. coli* BL21(DE3) and BLR(DE3), only minimal nanobody production was observed, barely distinguishable from plasmid-free controls. However, the cytoplasmic NB-associated band intensity in BL21(DE3) was found to be marginally higher in comparison to its periplasmic variants. Hence, as BL21(DE3) is a widely employed, traditionally T7-RNA-polymerase based production strain [44] it was designated as a reference strain for the following bioreactor studies. Conversely, BLR(DE3) was excluded on the basis of its comparable low expression levels and its close genetic similarity to BL21(DE3) as well as BL21(DE3) transformed with the periplasmic constructs.

*E. coli* XL1-Blue MRF′ exhibited detectable but weak nanobody production combined with poor growth performance, confirming its preferential use as a cloning host rather than a production system [45]. In contrast, *E. coli* NEB Express demonstrated robust growth and strong nanobody production for both periplasmic constructs (SP_OmpA_ and SP_PelB_), thus qualifying these strain-plasmid combinations as promising for further scale-up.

Both SHuffle^®^ T7 Express and SHuffle^®^ Express strains exhibited robust cytoplasmic NB expression, consistent with their genetically engineered ability to form cytoplasmic disulfide bonds [19]. Variants of the T7-based constructs that displayed lower periplasmic NB concentration were not pursued further, as the two SHuffle^®^ strains are specifically optimized for cytoplasmic production of disulfide bond containing recombinant proteins [19].

Despite the initial demonstration of robust production and favorable growth in shake flasks, *E. coli* Rosetta 2(DE3) strain was ultimately deemed unsuitable under fed-batch conditions due to the occurrence of pronounced substrate overflow inhibition, even at minimal feed rates (Supplementary Fig. S1). Consequently, it was excluded from further consideration in Sect. 3.3

Based on this qualitative screening, four host–plasmid systems (two for cytosolic production, one with SP_OmpA_ mediated and one with SP_PelB_ mediated secretion) as well as BL21(DE3) as reference (Table 5) were selected for subsequent characterization at the bioreactor scale.

### 3.3 Comparative characterization of five *E. coli-*plasmid combinations: Impact of subcellular targeting, strain background, and process conditions in 2 L fed-batch bioreactor cultivations

Next, a robust benchmark of five *E. coli*–plasmid combinations under strictly comparable, DoE-derived standard operating conditions was established (see Sect. 3.2; process control as described in Methods 2.3). Accordingly, the DoE-derived operating point was transferred without strain-specific re-optimization to enable direct comparison of the host-plasmid systems. This standardized benchmark does not imply that the selected conditions were individually optimal for every system. For the production phase, conditions were stringently standardized to evaluate host/construct differences under identical boundary conditions: induction with 100 µM of IPTG at OD_600 nm_ 150, temperature shift to 22.5 °C after induction, and cultivation for exactly 20 h of production, during which 400 mL of feed were added, so that all cultivations received the same substrate input during the production phase. Under these conditions, a fair comparison of product formation capacity and the derived performance metrics across the five host–plasmid systems was ensured.

Table 6 summarizes the key process parameters, as well as the endpoint values for biomass (cell dry weight, CDW) concentration, yield coefficients, and productivities derived from them. The comparison includes cytosolic localization of the NB (pT7-Cyto/ptac-Cyto) and signal-peptide-mediated export of the NB to the periplasm via Sec-dependent signal peptides of OmpA and PelB (ptac-OmpA/ptac-PelB), respectively. NB concentrations were again determined after whole-cell disruption and affinity purification.

**Table 6:** Key cultivation parameters and NB production metrics of five *E. coli*–plasmid combinations in 2 L high-cell-density fed-batch bioreactor cultivations

	SHuffle® T7 Express pT7-Cyto	NEBExpress® ptac-OmpA	NEBExpress® ptac-PelB	SHuffle® Express ptac-Cyto	BL21(DE3) pT7-Cyto
Cultivation replicates (n) [-]	3	2	2	3	3
OD_600nm_ at induction [-]	149.5 ± 4.0	146.0 ± 1.5	149.5 ± 5.0	140.7 ± 4.0	150.6 ± 13.1
Batch phase duration [h]	7.23 ± 0.05	7.70 ± 0.13	7.64 ± 0.19	6.77 ± 0.75	7.62 ± 0.89
Fed-batch phase (feed start to induction) [h]	14.75 ± 1.38	10.82 ± 1.08	10.83 ± 1.08	13.00 ± 0.39	13.68 ± 1.40
Total cultivation time [h]	41.98 ± 1.35	38.92 ± 0.54	38.47 ± 0.89	39.77 ± 1.68	41.43 ± 1.18
Total feed volume [mL]	637.7 ± 18.7	623.0 ± 17.0	621.5 ± 16.5	664.0 ± 14.2	652.7 ± 42.4
OD_600nm_ at harvest [-]	225.5 ± 11.0	228.2 ± 15.8	229.1 ± 10.9	224.6 ± 32.1	217.3 ± 30.6
CDW concentration at harvest [g L^−1^]	**64.54 ± 3.20**	**65.34 ± 4.59**	**65.58 ± 3.19**	**64.28 ± 9.37**	**60.10 ± 8.92**
Overall specific growth rate, µ [h^−1^]	0.194 ± 0.005	0.209 ± 0.004	0.212 ± 0.006	0.205 ± 0.012	0.196 ± 0.007
NB post-purification concentration at harvest [g L^−1^]	**2.19 ± 0.51**	**0.48 ± 0.03**	**2.43 ± 0.04**	**1.08 ± 0.34**	**0.06 ± 0.01**
Y_X/S_ (CDW/substrate) [g g^−1^]	0.203 ± 0.011	0.210 ± 0.020	0.210 ± 0.016	0.194 ± 0.033	0.190 ± 0.031
Y_P/X_ (NB/CDW) [g g^−1^]	0.034 ± 0.007	0.007 ± 0.000	0.037 ± 0.001	0.016 ± 0.003	< 0.001
Y_P/S_ (NB/substrate) [g g^−1^]	0.007 ± 0.001	0.002 ± 0.000	0.008 ± 0.000	0.003 ± 0.001	< 0.001
Volumetric productivity Pr (production phase) [g L^−1^ h^−1^]	**0.110 ± 0.026**	**0.024 ± 0.010**	**0.122 ± 0.002**	**0.054 ± 0.017**	**0.003 ± 0.000**
Overall space–time yield [g L^−1^ h^−1^]	0.052 ± 0.010	0.012 ± 0.001	0.063 ± 0.003	0.028 ± 0.010	< 0.001
Apparent biomass-specific productivity qP (production phase) [mg_NB_ g_CDW_^−1^ h^−1^]	1.69 ± 0.35	0.36 ± 0.01	1.85 ± 0.06	0.82 ± 0.14	0.05 ± 0.02

The production phase at 22.5 °C lasted 20 h with a total feed addition of 400 mL. Values are reported as mean of biological replicates (n) ± standard deviation

The duration of the batch phase ranged from 6.8 to 7.7 h, indicating good overall comparability, before feeding started. Further, across all systems, the OD_600nm_ at induction was closely aligned with the target value defined in the DoE (OD_600nm_ ≈ 150), ranging from 141 (SHuffle^®^ Express ptac-Cyto) to 151 (BL21(DE3) pT7-Cyto). More pronounced differences were observed in the time interval before, between the start of feeding and induction. The two NEB Express plasmid strains reached the OD required for induction after 10.8 h, while the other strains required between 13.7 and 14.8 h. Accordingly, the fed-batch phase prior to induction was approximately 2.2–3.9 h shorter for the NEB Express systems than for the other hosts, which is consistent with a higher apparent growth rate during this pre-production phase. The total process time ranged from 38.5 h (NEBExpress^®^ ptac-PelB) to 42.0 h (SHuffle^®^ T7 Express pT7-Cyto). The subsequent production phase is methodologically decoupled from the preceding phases, as it was carried out in a strictly identical manner for all cultivations (temperature shift to 22.5 °C; 20 h production with 400 mL feed, corresponding to 20 mL h^−1^).

A key control for comparability is the biomass at harvest. CDW concentrations were highly similar for four of the five systems with 64.3–65.6 g L^−1^ (NEBExpress^®^ ptac-OmpA/ptac-PelB, SHuffle^®^ T7 Express pT7-Cyto, SHuffle^®^ Express ptac-Cyto). BL21(DE3) pT7-Cyto had a lower CDW of 60.1 g L^−1^. Consistently, OD_600nm_ at harvest ranged from 217 (BL21(DE3) pT7-Cyto)—229 (NEBExpress® ptac-PelB). Thus, for the majority of host-plasmid-systems no growth-related limitation was evident. The largely comparable biomass formation supports the interpretation that differences in nanobody production primarily reflect strain-specific production efficiency rather than differences in available cell mass. Consequently, the final NB concentrations after affinity purification, reported per liter of culture broth at harvest, are directly comparable with concentrations of 0.06 g L^−1^ (BL21(DE3) pT7-Cyto), 0.48 g L^−1^ (NEBExpress^®^ ptac-OmpA), 2.43 g L^−1^ (NEBExpress® ptac-PelB), 2.19 g L^−1^ (SHuffle^®^ T7 Express pT7-Cyto), and 1.08 g L^−1^ (SHuffle^®^ Express ptac-Cyto).

To contextualize the endpoint concentrations, Table 6 reports selected performance indicators that enabled process benchmarking and quantify strain-specific differences in product formation: volumetric productivity (Pr, g L^−1^ h^−1^) as the primary process key performance indicator (KPI) and product yield on biomass (Y_P/X_, g g^−1^) as a biomass-normalized product formation metric. Product yield on substrate (Y_P/S_, g g^−1^) was used as an additional resource-related descriptor.

Over the 20 h production phase, NEBExpress^®^ ptac-PelB achieved the highest volumetric productivity (Pr = 0.122 g L^−1^ h^−1^). That Pr was slightly higher than that of SHuffle® T7 Express pT7-Cyto (0.110 g L^−1^ h^−1^). Both systems substantially exceeded SHuffle^®^ Express ptac-Cyto (0.054 g L^−1^ h^−1^) and NEBExpress^®^ ptac-OmpA (0.024 g L^−1^ h^−1^). BL21(DE3) pT7-Cyto remained near baseline at 0.003 g L^−1^ h^−1^. Because CDW was tightly clustered for four systems, Y_P/X_ is particularly informative to isolate strain-specific production performance: NEBExpress^®^ ptac-PelB (Y_P/X_ = 0.037 g g^−1^) and SHuffle® T7 Express pT7-Cyto (0.034 g g^−1^) showed the highest biomass-normalized product formation, followed by SHuffle^®^ Express ptac-Cyto (0.016 g g^−1^) and NEBExpress^®^ ptac-OmpA (0.007 g g^−1^).

As an additional resource-related descriptor, Y_P/S_ (NB yield on substrate) was compared for the two top systems: SHuffle^®^ T7 Express pT7-Cyto showed 0.007 g g^−1^, whereas NEBExpress^®^ ptac-PelB showed 0.008 g g^−1^. The very similar Y_P/S_ values, with a slightly higher value for NEBExpress® ptac-PelB, were consistent with the working hypothesis that in the SHuffle^®^ background additional cellular resources may be allocated to redox- and chaperone-assisted folding support, which could manifest as a subtly reduced net substrate-to-product conversion. This interpretation aligns with reports that intracellular nanobody production in *E. coli* can be limited by disulfide-dependent folding requirements and redox management and can therefore be improved by engineered redox control and/or folding catalysts [14, 21, 35].

Overall, the data underscore that process performance is not determined solely by the host orga-nism, but rather by the combination of host background and expression architecture (promoter/regulation, compartment/targeting design). This conclusion is supported by two observations: (I) For secretion to the periplasm within the same host organism (NEB Express), the choice of signal peptide had a dominant influence (SP_PelB_ ≫ SP_OmpA_) and (II) when using the identical plasmid (pT7-Cyto), product formation differed markedly between hosts (SHuffle® T7 ≫ BL21(DE3)). Both phenomena are consistent with the literature on host burden, limited folding capacity and translocation-related bottlenecks in highly driven expression systems [24, 28, 29, 36].

Regarding (I): The marked difference in performance between SP_PelB_ and SP_OmpA_, despite their identical target compartment (the periplasm), is compatible with SP-dependent differences in Sec-mediated translocation and processing as well as with previous reports indicating that secretion capacity can become the limiting factor under high recombinant protein production loads [22, 24–27]. In the context of the present data, this yields a process-relevant inference: targeting design is a first-order parameter for NB secretion under HCDC fed-batch conditions.

(II) The strongly reduced product formation of BL21(DE3) pT7-Cyto relative to SHuffle^®^ T7 Express pT7-Cyto, despite an identical expression architecture (T7 promoter; cytosolic targeting), supports a dominant host effect. SHuffle^®^ T7 Express is an enhanced derivative of BL21(DE3) carrying, among other modifications, mutations to enable the production of disulfide bond containing recombinant proteins. The required oxidizing conditions in the cytoplasm, necessary intracellular disulfide-bond formation, are generated due to the deletion of *trxB*, encoding thioredoxin reductase, and of *gor*, encoding glutaredoxin reductase, together with a mutation in the gene encoding the peroxiredoxin enzyme (ahpC*), in order to prevent the lethality of the first two mutations [46], as well as the intracellular production of the periplasmic disulfide bond isomerase DsbC, lacking its native signal sequence (Table 1, [19]). Based on this, plausible explanations include unfavorable resource allocation due to high T7-driven transcription/translation load after induction (“host burden”) and/or an increase in incorrectly folded NB resulting in a NB aggregation and proteolytic turnover that consumes cellular resources in BL21(DE3) [28, 29, 36]. No pre-induction expression was detected and antibiotic selection was maintained, arguing against substantial leakiness or plasmid loss as primary drivers.

**Fig. 5:**
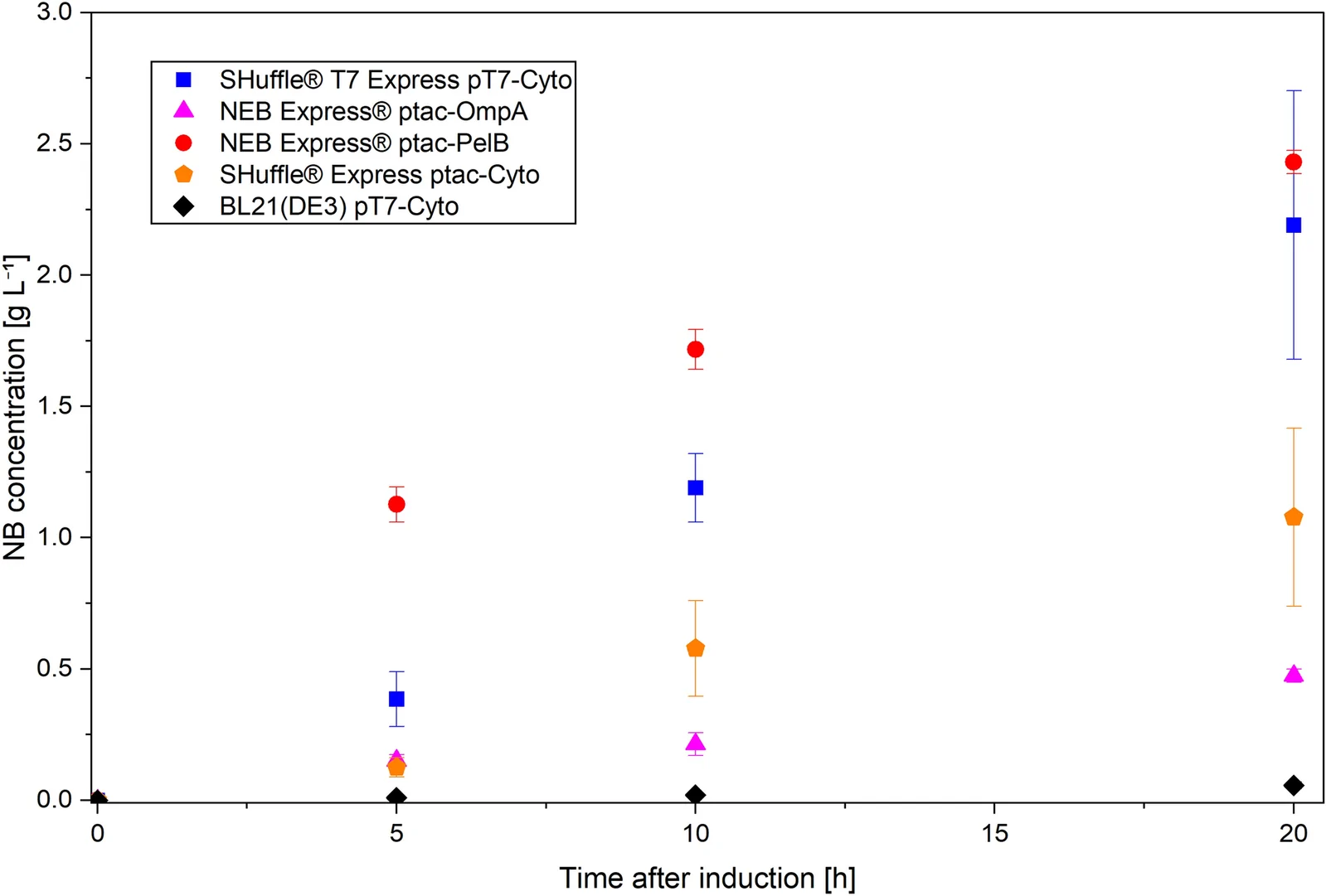
NB concentration over time for five *E. coli*–plasmid combinations in 2 L high-cell-density fed-batch cultivations. Samples were taken 5, 10, and 20 h after IPTG induction (100 µM IPTG, constant feed: 400 mL). Data are shown as mean ± SD of biological replicates

The key metrics derived in Table 6 are summarized in the time series of NB concentrations in Fig. 5.

Across all five host-plasmid combinations, NB concentrations increased approximately linearly within the sampled window and no plateauing trend was apparent toward the end of the 20 h production window, suggesting that under the chosen boundary conditions, no production-limiting saturation was reached. The observed near-linear production trajectory indicates that extending the fed-batch production phase could, in principle, provide additional potential for further titer increases. However, this would require experimental validation, as prolonged cultivation may become limited by oxygen transfer, nutrient availability, cell viability, plasmid stability, or increasing proteolytic and folding stress. The purified NB preparations generated from the benchmarked host-plasmid systems were subsequently subjected to product-quality assessment (Sect. 3.4).

Based on their superior production performance, NEBExpress^®^ ptac-PelB and SHuffle^®^ T7 Express pT7-Cyto were identified as the leading periplasmic and cytoplasmic production routes, respectively. These two systems were selected for follow-up bioreactor pilot studies, which are beyond the scope of the present manuscript.

### 3.4 Functional characterization of purified nanobodies

After process optimization and benchmarking of host-plasmid combinations, purified nanobodies were characterized to determine whether high volumetric productivity from cytoplasmic and periplasmic routes translated into comparable product quality and functionality. Therefore, apparent molecular mass, thermal unfolding behavior, and specific binding to the corresponding CD45-epitope were evaluated across the selected cytoplasmic and periplasmic production routes.

#### 3.4.1 Confirmation of molecular weight and proper processing of purified nanobodies

**Fig. 6:**
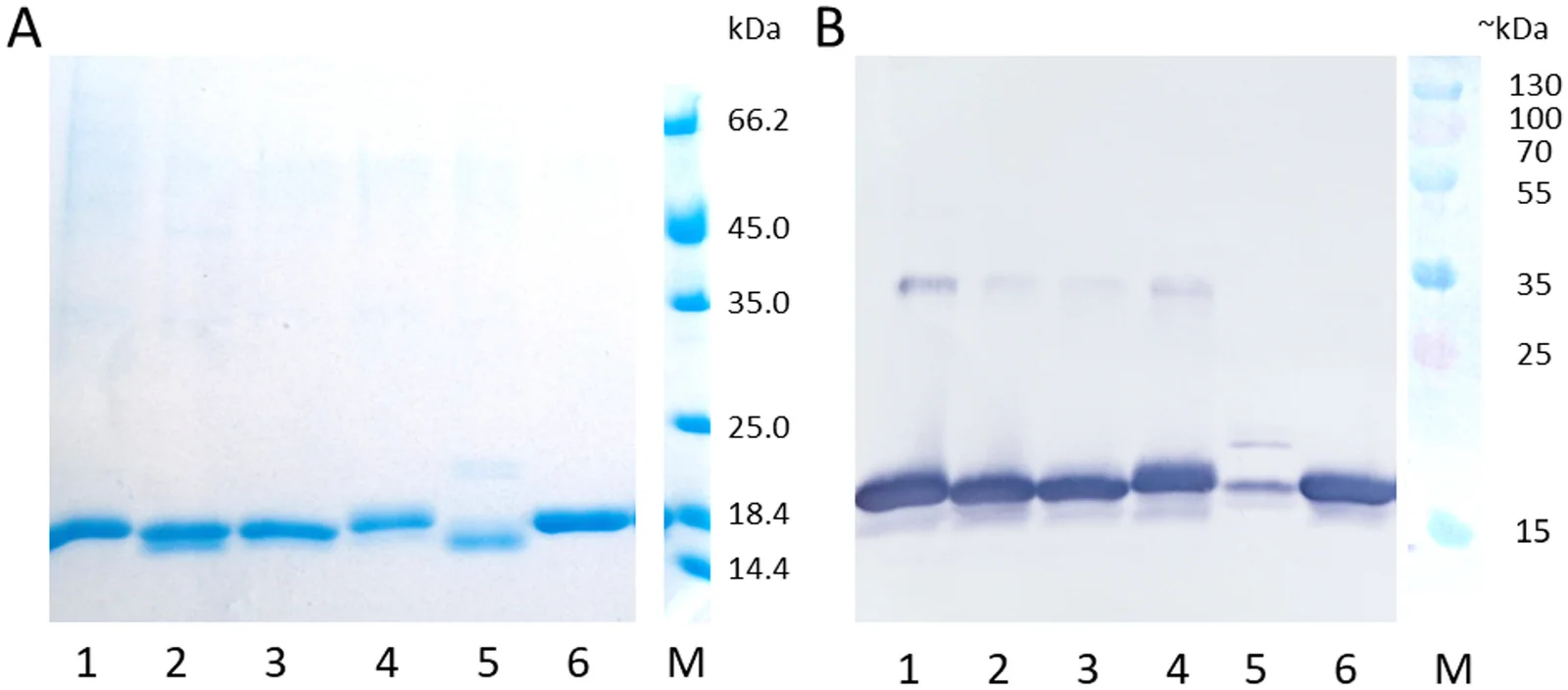
SDS-PAGE and Western blot analyses of purified anti-hCD45 nanobody. **A** Equal amounts of Strep-tag affinity-purified anti-hCD45 NB were loaded on an SDS-gel. Proteins were stained with Coomassie. M: Pierce Unstained Protein MW Marker (Thermo Fisher Scientific). **B** Equal amounts of Strep-tag affinity-purified anti-hCD45 NB were loaded on an SDS-gel. Proteins were blotted on PVDF membrane and immunodetected using Strep-Tactin® AP (iba Lifesciences GmbH, Göttingen, Germany). M: Prestained protein ladder (Fermentas). Lane 1: SHuffle® T7 Express pT7-Cyto; Lane 2: NEBExpress® ptac-OmpA; Lane 3: NEBExpress® ptac-PelB; Lane 4: SHuffle.® Express ptac-Cyto; Lane 5: BL21(DE3) pT7-Cyto; Lane 6: Commercial Strep-tagged anti-hCD45 NB (IBA Lifesciences GmbH, Göttingen, Germany)

To analyze the size of the recombinant nanobody confirming correct processing and production without proteolysis of anti-hCD45 NB, equal amounts of Strep-tag affinity-purified nanobody were analyzed by SDS-PAGE and Western blot. A commercial disulfide-containing anti-hCD45 NB produced cytoplasmically in SHuffle® T7 Express pT7-Cyto (IBA Lifesciences GmbH, Göttingen, Germany) served as the reference control (Fig. 6). This NB was located intracellularly after recombinant production, purified via Strep-tag affinity chromatography followed by size exclusion chromatography (iba Lifesciences GmbH, Göttingen, Germany). The last step was omitted in all purification steps within this study.

SDS-PAGE analysis revealed one dominant band in all lanes corresponding to a protein with a size between 14.4 and 18.4 kDa (Fig. 6 A). However, sizes appeared to differ slightly based on the SDS-gel. While the size of cytoplasmically located anti-hCD45 NB produced with SHuffle^®^ T7 Express pT7-Cyto and SHuffle^®^ Express ptac-Cyto equaled the size of the commercial anti-hCD45 NB, the sizes of the secreted anti-hCD45 NB were marginally smaller which is due to some missing amino acids at the N-terminus. After secretion, the N-terminal SPs (SP_OmpA_ and SP_PelB_) are cleaved leaving the N-terminus without the starting methionine and a few additional amino acids due to the cloning procedure of the plasmid used for cytoplasmic localization. The protein band representing the purified anti-hCD45 NB after production in BL21(DE3) pT7-Cyto indicated the smallest protein despite intracellular localization potentially indicating proteolytic degradation. The Western blot analysis based on detection of the C-terminal Strep-tag (Fig. 6 B). With this, all proteins could be detected hinting to an entire C-terminus. Again, the two secreted CD45 NB produced with NEBExpress^®^ ptac-OmpA and NEBExpress^®^ ptac-PelB had a minimally smaller size than the intracellularly located NB. The signal of the anti-hCD45 NB produced with BL21(DE3) pT7-Cyto was found to be very weak although the same amounts were loaded onto the corresponding gel. But, in contrast to the SDS-gel analysis, this band had the same size as all other bands. That strongly indicated that anti-hCD45 NB produced with BL21(DE3) pT7-Cyto was C-terminally degraded. In addition to the signals with a size of around 15 kDa, weak signals at approximately twice the molecular mass (34 kDa) were observed for proteins derived from SHuffle^®^ T7 Express pT7-Cyto, NEBExpress^®^ ptac-OmpA, NEBExpress^®^ ptac-PelB and SHuffle^®^ Express ptac-Cyto, suggesting the presence of small amounts of higher-molecular-weight NB, possibly dimeric nanobody forms. For BL21(DE3) pT7-Cyto, a weak additional band above the signal of the monomeric, full-length NB was detected in the Western blot, indicating that this protein also carried the Strep-tag which could not be explained. Due to the weak performance of BL21(DE3) pT7-Cyto no further analysis was conducted. No higher-molecular-weight signal was detected for the commercial anti-hCD45 NB, which may plausibly be attributed to the additional size-exclusion chromatography step applied after affinity tag purification of this NB (IBA Lifesciences GmbH, Göttingen, Germany).

#### 3.4.2 Analysis of conformational stability of purified anti-hCD45 nanobody

**Fig. 7:**
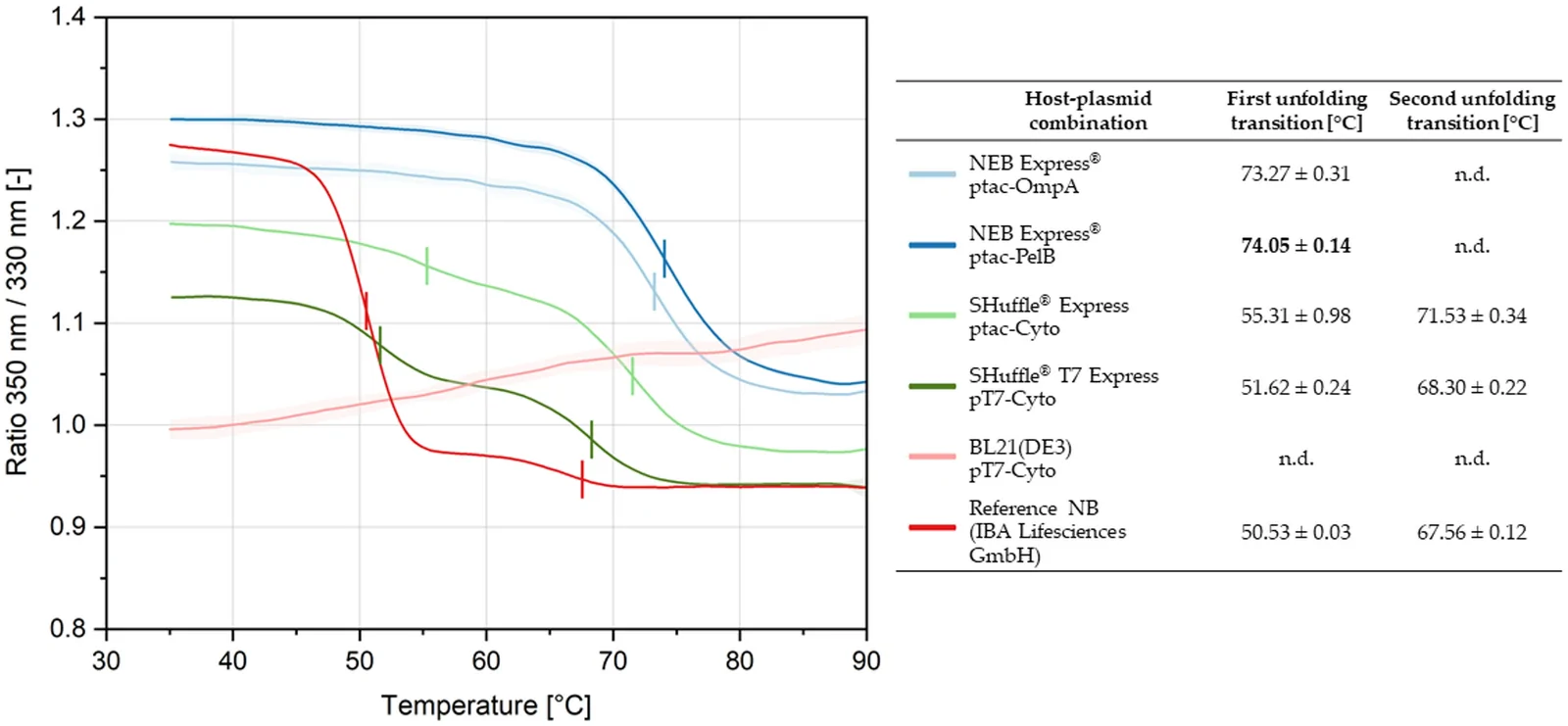
Thermal unfolding curves of purified CD45 NB samples determined by nanoDSF (Prometheus, NanoTemper Technologies GmbH, Munich, Germany). Intrinsic fluorescence was recorded as the 350/330 nm ratio over a temperature range between 30 and 90 °C. Changes in the fluorescence ratio reflect thermally induced conformational transitions and were used to assess the unfolding behavior of the purified NB samples. Vertical colored ticks indicate the determined unfolding events, corresponding to the inflection points of the respective curves. The corresponding first and second unfolding transition temperatures are summarized in the adjacent table. All samples were measured in PBS in technical triplicates; n.d., not detected

To further characterize the purified CD45 nanobodies, nanoDSF measurements were performed to assess their thermal stability and unfolding behavior. The analysis aimed to determine whether the produced nanobodies exhibited defined thermal unfolding transitions consistent with a properly folded conformation. Particular attention was given to whether the observed thermal stability was compatible with the formation of the characteristic intramolecular disulfide bond, which is expected to contribute to structural stabilization. The corresponding thermal unfolding curves are shown in Fig. 7.

As shown in Fig. 7, the nanoDSF analysis revealed distinct differences in the thermal unfolding behavior of the purified CD45 NB preparations. In all evaluable samples, with the exception of BL21 (DE3) pT7-Cyto, the fluorescence ratio remained largely stable at lower temperatures and subsequently changed significantly upon heating, indicating defined, thermally induced conformational transitions. These transitions may therefore reflect global unfolding of the disulfide-stabilized VHH fold [16, 17]. However, as nanoDSF does not directly assess disulfide-bond integrity, this interpretation remains hypothetical.

The commercial anti-hCD45 NB provided by IBA Lifesciences GmbH exhibited two distinct unfolding transitions at 50.53 ± 0.03 °C and 67.56 ± 0.12 °C. Notably, the CD45 NB produced with SHuffle^®^ T7 Express pT7-Cyto within this study representing the same general cytoplasmic production route as the commercial NB displayed a highly similar biphasic unfolding profile, with transitions at 51.62 ± 0.24 °C and 68.30 ± 0.22 °C. A comparable pattern was also observed for anti-hCD45 NB produced with SHuffle^®^ Express ptac-Cyto with unfolding transitions at 55.31 ± 0.98 °C and 71.53 ± 0.34 °C. Thus, all cytoplasmically produced NB that yielded evaluable nanoDSF signals demonstrated two thermally distinguishable transitions, with a first event in the range of approximately 50–55 °C and a second event at approximately 68 – 72 °C. In contrast, anti-hCD45 NB intracellularly produced with BL21(DE3) pT7-Cyto showed no clearly resolved unfolding transition (Fig. 7). Given the possible proteolytic degradation suggested by the SDS-PAGE and Western blot analyses (Fig. 6), the lack of a defined nanoDSF transition is consistent with the absence of a sufficiently pronounced and structurally coherent, NB-derived unfolding signal in the purified preparation.

The two NB secreted with NEBExpress^®^ ptac-OmpA and NEBExpress^®^ ptac-PelB showed only one single major unfolding transition which occurred at significantly higher temperatures of 73.27 ± 0.31 °C and 74.05 ± 0.14 °C, respectively. Compared with all cytoplasmic based NBs, these two NBs thus combined the highest apparent thermal stability with the most uniform unfolding behavior. Furthermore, the absence of a second, resolved transition suggests that the periplasmically produced NB preparations were more homogeneous under the tested conditions than their corresponding cytoplasmic variants.

Taken together, these data indicate that periplasmic NB production was associated with a more stable and more uniform nanobody population than cytoplasmic production in the strain panel adapted for intracellular disulfide bond formation (Table 1, [19]) investigated here. This interpretation is mechanistically plausible, because secretion into the *E. coli* periplasm exposes the recombinant protein to a native oxidizing environment in which the Dsb proteins naturally support disulfide bond formation and oxidative folding [47], whereas cytoplasmic nanobody expression is generally challenged by the reductive intracellular environment, which can compromise correct disulfide bond formation and folding homogeneity. Importantly, even though SHuffle-derived strains are specifically engineered generating an oxidizing cytoplasm to improve disulfide bond formation (Table 1; [19]), the present data still indicate a broader distribution of nanobody species in the cytoplasmic samples, reflected by the reproducible occurrence of two unfolding transitions (Fig. 7). This suggests that the chaperone- and redox-assisted cytoplasmic folding environment was sufficient to generate detectable and partially stable nanobody in contrast to non-adapted BL21(DE3), but did not result in the same degree of conformational uniformity as observed for the secreted NB. Such an interpretation is in line with the general observation that cytoplasmic expression of disulfide bond-dependent proteins remains more challenging and can still lead to misfolding or heterogeneous folding states despite dedicated folding-support systems [19, 21, 35].

Overall, the nanoDSF data identify the secretion to the periplasm as the most favorable route among the tested production systems with respect to thermal stability and folding uniformity of anti-hCD45 nanobody. Within this study, both periplasm-based NBs indicated a single high-temperature unfolding transition above 73 °C whereas all evaluable cytoplasmic based NBs showed lower and biphasic transitions (Fig. 7). Consequently, the results strongly support the conclusion that secretion to the periplasm promoted the formation of a more homogeneous and thermally robust anti-hCD45 nanobody compared to cytoplasmic production, making it the preferable production route from a product-quality perspective.

#### 3.4.3 Target binding to hCD45-positive cells analyzed by flow cytometry

Following production, purification and biophysical characterization, the antigen-binding functionality of purified anti-hCD45 NB recombinantly produced with SHuffle^®^ T7 Express pT7-Cyto, NEBExpress^®^ ptac-OmpA, NEBExpress^®^ ptac-PelB and SHuffle^®^ Express ptac-Cyto was evaluated using flow cytometric immunofluorescence on CD45-positive Jurkat cells (Table 7). The BL21(DE3)-derived nanobodies were not included in the flow cytometric binding analysis due to the insufficient concentration (Table 7).

**Table 7:** Flow cytometric immunofluorescence analysis of CD45-specific NB binding to Jurkat ACC-282 cells

Sample ID	Cell-gated events [events]	Single-cell [% parent]	Q1-UR [% parent]	Median PE-A [arbitrary units]	Fold over background [-]
SHuffle® T7 Express pT7-Cyto	2241.5 ± 61.5	89.29 ± 2.45	**99.98 ± 0.02**	1,627,326	269.10
NEB Express® ptac-OmpA	2363.0 ± 31.0	84.65 ± 1.11	**99.93 ± 0.03**	853,588	141.10
NEB Express® ptac-PelB	2582.5 ± 270.5	78.31 ± 8.20	**99.93 ± 0.07**	1,315,724	217.50
SHuffle® Express ptac-Cyto	2572.5 ± 1.5	77.75 ± 0.05	**99.75 ± 0.15**	1,445,489	239.00
Reference NB (IBA Lifesciences GmbH)	2190.5 ± 474.5	71.68 ± 3.37	**99.98 ± 0.02**	1,129,942	186.80
Commercial mAb (IBA Lifesciences GmbH)	2599.5 ± 3.5	76.94 ± 0.11	**20.73 ± 0.87**	10,610	1.75
Background	2536.0 ± 48.7	78.89 ± 1.53	**0.12 ± 0.02**	6,048	1.00

Bound NB (136 ng_NB_/1 × 10^6^ cells) were detected using R-Phycoerythrin (PE)-conjugated Strep-Tactin®XT (iba Lifesciences GmbH, Göttingen, Germany). A PE-labeled monoclonal mouse anti-hCD45 antibody (anti-hCD45 mAb) (1 µg_mAb_/1 × 10^6^ cells) was included as a reference control for CD45 staining. Unstained cells and background controls consisting of Jurkat cells incubated with PE-conjugated Strep-Tactin®XT without the addition of NB served as negative controls. Samples were analyzed on the CytoFLEX flow cytometer (Beckman Coulter, Brea, CA, USA). Data were evaluated after scatter-based gating to exclude debris and aggregates, followed by analysis of 2,000 single-cell events per sample. “Q1-UR” denotes the upper-right quadrant in the fluorescence dot plot and represents the double-positive cell population after quadrant gating. The “median PE-A” value represents the median phycoerythrin fluorescence intensity of the single-cell population. The “Fold over background” was calculated by normalizing the mean “median PE-A” of each sample to the mean “median PE-A” of the background control. Data are shown as mean ± SD of 3 technical replicates

All analyzed purified anti-hCD45 NB resulted in nearly complete staining of the Jurkat cell population. The proportion of Q1-UR (double-positive events, Table 7) across the various NB preparations ranged from 99.75 ± 0.15 % to 99.98 ± 0.02 %. In contrast, the background control, consisting of Jurkat cells incubated with PE-conjugated Strep-Tactin^®^XT in the absence of NB, remained essentially negative with only 0.12 ± 0.02 % double-positive events. This clear separation between NB-treated samples and the background signal from the detection reagents indicates that the observed fluorescence signal depended on the binding of the Strep-tagged anti-hCD45 NB.

Since the staining of the Jurkat cells caused by the anti-hCD45 NB binding was nearly complete independently of the nanobody preparation, the median PE-A fluorescence intensity of the single-cell population was additionally used to compare the signal amplitude between samples. All nanobody preparations showed markedly increased fluorescence intensities relative to the background control. The highest signal was observed for the nanobody produced with SHuffle^®^ T7 Express pT7-Cyto, reaching a 269.1-fold increase over background. High fluorescence signals were also obtained for SHuffle^®^ Express ptac-Cyto, NEBExpress^®^ ptac-PelB, and the commercial anti-hCD45 nanobody, with 239.0-, 217.5-, and 186.8-fold increases over background, respectively. The detection with the anti-hCD45 produced with NEBExpress^®^ ptac-OmpA yielded the lowest fluorescence intensity, with a 141.1-fold increase over background. Nevertheless, it still detected virtually the entire target-cell population and remained clearly separated from the negative control.

A PE-labeled monoclonal mouse anti-human CD45 antibody (anti-hCD45 mAb) was included solely as a qualitative staining control to confirm the hCD45-positive phenotype of the Jurkat ACC-282 cells. Under the applied staining conditions, the mAb produced a detectable CD45-specific fluorescence signal, confirming that the target-cell population was accessible for antibody-based CD45 detection.

The BL21(DE3) pT7-Cyto-derived NB preparation was not included in the flow-cytometric binding analysis because the available amount and purity of this preparation were insufficient for reliable concentration adjustment and comparable staining. This is consistent with the weak target-protein signal observed by SDS-PAGE and Western blot and the absence of a clearly resolved nanoDSF unfolding transition for this sample.

In summary, the flow-cytometric immunofluorescence data demonstrate that all analyzed purified anti-hCD45 NB preparations, irrespective of cytoplasmic or periplasmic production, retained functional binding to hCD45-positive Jurkat cells after recombinant production and purification. Importantly, the same purified NB preparations were used for nanoDSF and flow-cytometric analysis within a two-day time frame, enabling direct comparison between thermal unfolding behavior and retained cell-binding activity. The combination of nearly complete staining of the target-cell population and negligible staining in the background control using the detection reagent alone provides strong evidence of NB-dependent cellular target binding. Differences in median PE-A intensity indicate variations in fluorescence signal amplitude among the preparations. However, the uniformly high proportion of Q1-UR double-positive events suggests that all analyzed NB preparations retained CD45-binding activity under the applied assay conditions.

## 5 Conclusions

This study establishes a controlled high-cell-density fed-batch process for the production of a functional anti-hCD45 nanobody with *E. coli* and demonstrates that gram-per-liter titers can be achieved under defined bioreactor conditions. The comparative evaluation showed that nanobody production performance was governed by the combined effects of host background and subcellular targeting design rather than by the host strain alone. Product-quality analysis revealed a clear route-dependent difference: periplasmic production generated nanobody preparations with higher apparent thermal stability and conformational homogeneity whereas cytoplasmic SHuffle^®^-derived preparations displayed biphasic unfolding behavior. Flow cytometric analysis with Jurkat cells confirmed that the recombinant nanobodies have CD45-specific binding activity, linking high volumetric production to retained functional quality.

Taken together, these findings show that efficient nanobody production in *E. coli* requires coordinated optimization of induction strategy, production temperature, host background, and subcellular targeting. Within the tested host-plasmid systems, signal-peptide-mediated periplasmic targeting provided the most favorable overall production profile, yielding the highest total recoverable NB concentration and generating NB preparations with greater apparent thermal stability and conformational homogeneity than those obtained from redox-engineered cytoplasmic SHuffle® systems. The process therefore provides a bioreactor-based platform for scalable production of functional nanobodies and supports rational selection of production routes based on titer and product quality. Based on the production and quality profiles obtained in this study, NEBExpress^®^ ptac-PelB and SHuffle^®^ T7 Express pT7-Cyto were selected as the most promising periplasmic and cytoplasmic routes, respectively, for future pilot-scale transfer studies.

## Supplementary Information

Supplementary Material 1.

Supplementary Material 2.

## Data Availability

All datasets generated and/or analyzed during the current study, as well as the associated code used for statistical modelling, are available from the corresponding author on reasonable request.

## Abbreviations

CD45: Cluster of differentiation 45
CDW: Cell dry weight
CWW: Cell wet weight
DoE: Design of experiments
DO: Dissolved oxygen
EDTA: Ethylenediaminetetraacetic acid
HCDC: High-cell-density cultivation
IPTG: Isopropyl β-D-1-thiogalactopyranoside
Kan^R^: Kanamycin resistance
KPI: Key performance indicator
NB: Nanobody
nanoDSF: Nano differential scanning fluorimetry
OD_600nm_: Optical density at 600 nm
OmpA: Outer membrane protein A
PBS: Phosphate-buffered saline
PE: Phycoerythrin
PE-A: Phycoerythrin fluorescence area
PelB: Pectate lyase B
Pr: Volumetric productivity
pT7: T7-promoter-based expression plasmid
ptac: Tac-promoter-based expression plasmid
Q1-UR: Upper-right quadrant
RSM: Response surface methodology
SD: Standard deviation
SDS-PAGE: Sodium dodecyl sulfate–polyacrylamide gel electrophoresis
Sec: General secretory pathway
SRP: Signal recognition particle
STBR: Stirred-tank bioreactor
T7: Bacteriophage T7 promoter/RNA polymerase expression system
TB: Terrific Broth
Tris–HCl: Tris(hydroxymethyl)aminomethane hydrochloride
UV: Ultraviolet
VHH: Variable domain of camelid heavy-chain-only antibodies
Y_P/S_: Product yield on substrate
Y_P/X_: Product yield on biomass
